# A case for seeking sex-specific treatments in Alzheimer’s disease

**DOI:** 10.3389/fnagi.2024.1346621

**Published:** 2024-02-13

**Authors:** Marina A. Lynch

**Affiliations:** Trinity College Institute of Neuroscience, Dublin, Ireland

**Keywords:** Alzheimer’s disease, sex, clinical trials, inflammation, amyloid

## Abstract

There is no satisfactory explanation for the sex-related differences in the incidence of many diseases and this is also true of Alzheimer’s disease (AD), where females have a higher lifetime risk of developing the disease and make up about two thirds of the AD patient population. The importance of understanding the cause(s) that account for this disproportionate distribution cannot be overestimated, and is likely to be a significant factor in the search for therapeutic strategies that will combat the disease and, furthermore, potentially point to a sex-targeted approach to treatment. This review considers the literature in the context of what is known about the impact of sex on processes targeted by drugs that are in clinical trial for AD, and existing knowledge on differing responses of males and females to these drugs. Current knowledge strongly supports the view that trials should make assessing sex-related difference in responses a priority with a focus on exploring the sex-stratified treatments.

## Background

Almost 65% of AD patients are women and the increased risk of AD in women is present even when their longer lifespan is taken into account (Viña and Lloret, 2010). A recent review concluded that, once a diagnosis is made, clinical symptoms occur more rapidly in females (Podcasy and Epperson, 2016) with greater tau pathology in females compared with males (Luchsinger et al., 2020; Edwards et al., 2021).

The evidence related to sex differences in Aβ deposition in AD is less clear but there is some evidence that there is a modest increase in Aβ pathology in females which may be age-related (Oveisgharan et al., 2018; Luchsinger et al., 2020). Dystrophic, iron-containing microglia develop and cluster around Aβ plaques (Meadowcroft et al., 2015) and are more prevalent in tissue from female compared with male AD patients (O’Neill et al., 2022). The advent of RNA sequencing (RNAseq) technology, and particularly single nucleus RNAseq, has permitted the comparison of differentially-expressed genes in postmortem samples from AD patients and evidence indicates that there is an upregulation of genes transcripts reflecting inflammatory/immune processes particularly in samples from female AD patients (Paranjpe et al., 2021; Guo et al., 2022), consistent with the knowledge that several genes involved in immune regulation are located on the X chromosome. One has to consider the importance of this given that about 20% of genes from the inactive X chromosome escape inactivation, including genes that play a role in inflammation and/or impact on AD risk (Youness et al., 2021; Song et al., 2024). However several genes associated with inflammation/the immune response were also upregulated in cortical samples from control postmenopausal, compared with premenopausal, women (Sárvári et al., 2012; Coales et al., 2022), adding credence to the hypothesis that the triad of reduced ovarian function, inflammatory change and age amplifies AD risk and drives progression of disease in females (Mishra and Brinton, 2018).

The evidence that metabolism is also disrupted in AD is compelling (Demetrius et al., 2021) and several groups have reported decreased glucose utilization (Kapogiannis and Mattson, 2011; Butterfield and Halliwell, 2019; Wang et al., 2020) and mitochondrial dysfunction (Swerdlow, 2018; Wang et al., 2020; Misrani et al., 2021). Data from a longitudinal study over 2 years suggested that metabolic changes assessed by fluorodeoxyglucose-PET occurred to a greater extent in female AD patients compared with males (Park et al., 2023).

This review will consider that, at the very least, these sex-related differences in AD in pathology, immune function and metabolism point to the importance of interrogating the potential treatment regimes separately in males and females. It is important to point out that the majority of the studies cited here were carried out on western European and US populations; this is an acknowledged limitation that has been identified as a significant shortcoming in clinical trials (Reardon, 2023b).

## Risk factors and AD: interaction with sex

### Non-genetic risk factors in AD

The current estimate is that the risk of AD is reduced by about 40% if modifiable risk factors including cardiovascular disease, obesity and diabetes were eliminated (Livingston et al., 2020). Recent reviews (Mielke, 2018; Rahman et al., 2019) pinpoint a sex dimension in several risk factors (see Table 1) including level of education and social interaction, smoking, alcohol consumption and stress may also have a sex-related component (Podcasy and Epperson, 2016; Yan et al., 2018).

**TABLE 1:** Modifiable risk factors that are impacted by sex.

Modifiable risk factor	The sex dimension
Education	Poor education is reported to present a greater risk of AD in women (Letenneur et al., 2000) and risk of AD is reduced with additional years of education (Nebel et al., 2018). A large prospective cohort study of individuals born between 1930 and 1955 indicated a positive effect of education in women on fluency but not memory (Bloomberg et al., 2021).
Hearing loss	A study of 655 individuals found that moderate/severe hearing loss was associated with poorer cognitive function in men (Huang et al., 2020) although the risk of dementia was greater in older women (> 65 years) with sudden hearing loss (Tai et al., 2021).
Head trauma	A study of >14,000 individuals with head injury revealed that the risk of dementia was greater in women with head injury compared with men (Schneider et al., 2021).
Cardiovascular disease	A population-based study of >500,000 individuals found that women with cardiovascular disease are more likely to have AD than men with cardiovascular disease (Dong et al., 2022).
Alcohol	A recent metanalysis of 7 studies that included sex as a risk factor for AD did not identify clearcut differences between men and women although AD cases in European countries that could be attributed to alcohol were greater in men than women (Kilian et al., 2023).
Obesity	Among 6,582 non-demented individuals, 7% developed dementia within a 15 year follow-up. Obesity increased risk of dementia and was significantly greater in obese women (Ma et al., 2020).
Smoking	The risk of dementia is greater in women who smoke/have smoked (Zhang et al., 2021).
Depression	Untreated depression in women is associated with the likelihood of developing AD and treatment reduces this risk (Norton et al., 2019).
Social Isolation	Many studies suggest that social isolation poses a greater risk of dementia in women but a recent comprehensive review indicates a lack of consensus (Ren et al., 2023)
Physical inactivity	Evidence suggesting a sex-related difference in risk arising from physical inactivity is mixed with no clear association (Sindi et al., 2021)
Diabetes	Two studies with >85,000 (Lee et al., 2021) or >3000 (Verhagen et al., 2022) participants with type 2 diabetes established that during a 6 year follow-up period development of AD or accelerated cognitive decline was greater in women.
Air pollution	The incidence of AD is increased in individuals exposed to higher PM_2.5_ and NO_2_ (Shi et al., 2021). Some evidence suggests that air pollution has a greater negative impact on cognition in females (Kim et al., 2019). In a rat model of AD, exposure to traffic-related air pollution induced earlier and more profound amyloid pathology in females compared with males (Patten et al., 2021)
Inflammation	Data from a longitudinal study with >38,000 individuals found that *APOE*-ε4 carriers and females with higher CSF TNF-α, IL-9, and IL-12p40 levels were at the highest risk of progression to MCI/AD (Contreras et al., 2022).

Exercise and diet are among the strategies that can have a far reaching impact on a number of modifiable AD risk factors. A study of 44,000 individuals with a 10 year follow-up period provided convincing evidence that disabling dementia in both men and women was inversely proportional to daily physical activity (Ihira et al., 2022) but both aerobic and resistance training appear to improve executive function to a greater extent in women (Barha et al., 2017). Moreover, the association between physical and cognitive ability and memory reserve is stronger in women than men and this is influenced by *APOE4* (Pa et al., 2022). While a scientific basis for this remains to be determined, evidence from animal studies suggests that sex-related differences in exercise-stimulated growth factors, brain architecture and/or cardiac or respiratory responses may play a role (Barha and Liu-Ambrose, 2018).

Adiposity is a confirmed risk factor for AD (Deng et al., 2022) and some evidence points to it driving inflammation to a greater extent in females compared with males (Khera et al., 2009) although gray matter volume decreases to a greater extent in obese males compared with females, suggesting that obese males may be at greater risk of later cognitive decline (Taki et al., 2008). Diet also appears to have sex-dependent effects with evidence that adherence to a Mediterranean diet exerts a greater benefit (Gregory et al., 2022), and greater protection against AD (Rahman et al., 2019), in women.

The evidence is that menopause constitutes a risk of AD and it has been reported that the reduction in brain glucose metabolism that occurs with the menopause is linked with prodromal AD and, indeed, it has been proposed that early estrogen therapy may be a useful strategy in reducing the menopause-associated risk of AD (Mosconi and Brinton, 2018; Mosconi et al., 2018; Mishra et al., 2022).

Despite heterogeneity in evaluating educational achievement, a recent review concluded that the evidence linking poorer education and increased risk of AD was robust (Maccora et al., 2020). The fact that education was more limited for women than men in the past opened up the possibility that this contributed to the greater risk of AD in women (Bloomberg et al., 2021; Gong et al., 2023) and a recent analysis of 2 prospective studies has suggested that females may benefit more from education than men in terms of AD risk (Bloomberg et al., 2021).

### Genetic risk factors in AD

*APOE* is the greatest genetic risk factor for sporadic AD and the ε4 allele confers the greatest risk (Corder et al., 1993; Strittmatter et al., 1993) although many additional susceptibility genes, particularly related to amyloid, tau, immunity, lipids and endocytosis, have been identified by Genome-wide association studies (GWAS) (Bellenguez et al., 2022). Genes that confer significant risk of AD include *TREM2*, *BIN1*, *PICALM*, *CLU* and *CR1*.

#### The interaction between sex and APOE

The risk of AD in *APOE4*-ε4 carriers has been reported to be greater in females (Riedel et al., 2016) but recent evidence indicated that having 1 copy of *APOE4*-ε4 was associated with similar risk of AD in men and women between the ages of 55 and 85 years, whereas a greater risk was observed in women aged 65–75 years (Neu et al., 2017). *APOE4* is associated with faster disease progression (Buckley et al., 2018), increased tau pathology (Deming et al., 2018) and more rapid decline in performance in memory tasks (Ungar et al., 2014) in female carriers (Neu et al., 2017). It is a predictor of transition from MCI to AD in males and females, but with a greater impact in females. In preclinical studies, memory impairment was observed at an earlier age in female *ApoE* 3xTg mice, and amyloid pathology and β-secretase expression were more pronounced in the hippocampus of female mice (Hou et al., 2015).

The impact of hormone replacement therapy on AD risk in postmenopausal women is inconsistent and may be related to *APOE4* since it was reported to improve episodic memory (Burkhardt et al., 2004) and reduced the risk of cognitive impairment (Yaffe et al., 2000) albeit only in *APOE-ε4* non-carriers.

#### Interactions between sex and other genes that confer risk

A number of studies have reported transcriptome-wide sex differences in AD including genes that suggested activation of the immune system which were increased in male and female AD patients although the greater number of dysregulated genes, for example *CHI3L1* (Sanfilippo et al., 2019), was markedly increased in samples from females (Paranjpe et al., 2021). Evidence from the APP/PS1 mouse model also revealed female-specific upregulation of genes that modulate inflammatory mediators (Manji et al., 2019; Guillot-Sestier et al., 2021). Several other sex-related differences in genes have been identified and some are listed in Table 2.

**TABLE 2:** Sex-related differences in some genes that confer AD risk.

Gene	Link to AD	References
*CXCR4*	Female-specific increase in AD	Brooks and Mias, 2019
*SERPINB1, SERPINB6, SERPINB9*	Correlation with amyloidosis in females	Deming et al., 2018
*OSTN, CLDN16*	Correlation with tau pathology in females	Deming et al., 2018
*NCL,KIF2A*	Associated with tau phosphorylation in females	Cáceres and González, 2020
*BIN1, MS4A6A, DNAJA2, FERMT2*	Contribute to AD progression more in females than males. May confer a higher risk in females.	Fan et al., 2020
*ACE*	An association between ACE genotype and AD reported in females	Crawford et al., 2000
*BDNF*	Female-specific polymorphism (rs6265) is associated with susceptibility to AD	Li et al., 2017
*ZBTB7C*	A variant (rs1944572) conferred an increase in AD risk in females and a decreased risk in males	Prokopenko et al., 2020
*RELN*	Polymorphisms (rs528528 and rs607755) are associated with AD risk in males	Fehér et al., 2015
*GRN*	Genetic variability is associated with AD risk in males	Viswanathan et al., 2009

Enrichment of genes reflecting upregulation of the PI3K-Akt signaling pathway was observed in brain samples from males and females but upregulation of genes related to the MAPK signaling pathway occurred in males only, which might translate into sex-specific responses to p38 MAPK inhibitors that are being assessed for efficacy in AD (Lee and Kim, 2017).

## Is there evidence of sex-related differences in response to approved drugs for AD?

Drug efficacy is impacted by hormones (Kwon et al., 2017) and while the emphasis may have been on assessing interactions between contraceptive hormones or stress hormones and drugs, evidence points to other interactions with sex hormones, for example between hormones and antiepileptic drugs (Taubøll et al., 2021), hormones and pain medication (Athnaiel et al., 2023), hormones and antidepressants (Pavlidi et al., 2021). Further study is likely to uncover other hormone:drug interactions, perhaps relevant to AD. Here the emphasis is on assessing the impact of sex on drug action in AD.

Five drugs and two monoclonal antibodies have been approved for the treatment of AD (Table 3).

**TABLE 3:** Drugs approved for the treatment of AD.

Drug and date approved	Target	Notes	The sex dimension
Donepezil (Aricept) 1996	Reversibly inhibits AChE; increases ACh availability enhancing cholinergic transmission	Does not modify the underlying pathophysiology of AD. Delays the progressive worsening of cognitive symptoms of AD. Modest effect. >250 clinical trials.	Variable data on sex-related changes in cognition including reports of no sex-related differences of donepezil (Canevelli et al., 2017), better effects in male AD patients (Davis and Barrett, 2009) and better effects in women AD patients (Scacchi et al., 2014)
Galantamine 2000	Weakly inhibits AChE and potentiates nicotinic and muscarinic receptors.	Benefits in global clinical function, behavioral symptoms, and activities of daily living.	Assessment of the impact of donepezil, rivastigmine and galantamine reported a more beneficial effect of cholinesterase inhibitors on cognition in males but the impact of the individual drugs was not reported (Wattmo et al., 2011).
Rivastigmine 1997	Reversible inhibitor of AChE and butyrylcholinesterase.	Dose-dependent effect on cognition, function, and activities of daily living.	Reduced cognitive decline in women AD patients treated (Scacchi et al., 2014). Delayed progression from MCI to AD in women (Ferris et al., 2009).
Tacrine	Reversible AChE inhibitor.	Largely replaced by other AChE inhibitors because of liver toxicity	Tacrine improved cognitive function to a greater extent in male, compared with female, e4 carriers (MacGowan et al., 1998).
Memantine 2002	NMDA antagonist (non-competitive, low- to medium-affinity). Also acts as an antagonist at 5HT3 and nicotinic (low-affinity) receptors.	Improves delusions, hallucinations, agitation, aggression, and irritability.	No published data on sex-related differences in efficacy. Women report difficulties with palatability (Ruiz et al., 2019).
Aducanumab June 2021	IgG1 monoclonal antibody against a conformational epitope on Aβ. Binds specifically to aggregated forms of Aβ. Recognizes Aβ plaques.	Approved by FDA (Accelerated approval pathway). Not approved by the European Medicines Agency, December 2021	Studies report benefit of aducanumab but sex differences have not been reported (Budd Haeberlein et al., 2022). An FDA report indicates no sex differences https://www.fda.gov/media/143577/download
Lecanemab July 2023	IgG1 monoclonal antibody. Binds with high affinity to Aβ soluble protofibrils.	Approved by FDA. Not yet approved by the European Medicines Agency.	Benefits of lecanemab were greater in men than women (van Dyck et al., 2023) or ineffective in women (Kurkinen, 2023).

### Is the response to FDA-approved drugs sex-dependent?

Sex-related differences in the responses of AD patients to cholinesterase inhibitors have been reported but data are limited with few studies explicitly analyzing the impact of sex on treatment outcomes as pointed out in recent reviews (Canevelli et al., 2017; Mehta et al., 2017). Mehta et al. (2017) evaluated 33 studies in which sex was considered as a demographic variable, sex-stratified data were reported in only 1 study where no difference was identified. Canevelli et al. (2017) reported that only 2 out of the 48 randomly-controlled trials specifically assessed sex differences and these reported no sex-related differences in cognition in response to donepezil, corroborating the findings of an earlier review which concluded that sex exerted a minimal impact on the effects of cholinesterase inhibitor efficacy in spite of evidence from animal studies (Haywood and Mukaetova-Ladinska, 2006).

The studies that analyzed the effect of sex are mostly small in scale and date back >10 years. One of the more recent studies assessed the effect of donepezil, rivastigmine and galantamine in a 3-year prospective study and reported that the decline in cognitive function, at least in the short term, was slower in cholinesterase inhibitor-treated individuals compared with controls and that efficacy was better in older persons and non ε4 carriers. Overall the response was better in males than females but data were not stratified and whether sex differences were observed for all 3 drugs is unclear (Wattmo et al., 2011).

Earlier investigations reported that donepezil-treated male AD patients performed better in the Boston Naming Test than females (Davis and Barrett, 2009), supporting earlier findings that reported greater efficacy of cholinesterase inhibitors in male AD patients (MacGowan et al., 1998). In contrast, others observed that women AD patients responded better to donepezil and rivastigmine than men, exhibiting less marked cognitive decline (Scacchi et al., 2014), and that rivastigmine delayed progression from MCI to AD in females but not males (Ferris et al., 2009). These inconclusive findings are reflected in data from preclinical studies where donepezil preferentially improved cognitive function in male rhesus monkeys (Buccafusco et al., 2003) while rivastigmine antagonized scopolamine-induced spatial memory to a greater extent in female rats (Wang et al., 2000).

Variables that may contribute to a sex difference in response to these drugs include the influence of sex hormones and sex-related differences in the cholinergic system (Yoshida et al., 2000; Moen and Lee, 2021; Bennett et al., 2022). *APOE* genotype may also exert an impact. It was shown that female, but not male, ε2/ε3 carriers with mild-to-moderate AD responded better to tacrine than ε4 carriers (Farlow et al., 1998) and that tacrine improved cognitive function in male ε4 carriers more than male non-carriers and female ε4 carriers (MacGowan et al., 1998), but others reported that while ε4 carriers responded better to tacrine, there was no impact of sex (Rigaud et al., 2000). The therapeutic response to donepezil may also be affected by *APOE* genotype with evidence suggesting that ε4 carriers respond better (Choi et al., 2008); the effect of sex was not reported in this study.

To my knowledge, sex-related differences in efficacy to memantine have not been studied although palability issues have been reported in women (Ruiz et al., 2019). Analysis in 3xTg mice revealed that 3 months treatment with memantine increased plasma concentration and decreased Aβ oligomers to a greater extent in females than males but improved cognition and decreased tau and Aβ plaques similarly in male and female mice (Martinez-Coria et al., 2010).

### Monoclonal antibodies that target amyloid

Aducanumab, an Aβ-directed monoclonal antibody that binds to aggregated Aβ and recognizes amyloid plaques in brain, was the first immunotherapy that received US Food and Drug Administration (FDA) approval. Sex-related differences were not described in several published papers/commentaries that reported on the findings in the aducanumab trials ENGAGE or EMERGE (Knopman et al., 2021; Kuller and Lopez, 2021; Sabbagh and Cummings, 2021; Budd Haeberlein et al., 2022; Schneider, 2022; Rahman et al., 2023) even though policy suggests that appropriate recognition is given to this issue.

Lecanemab, which selectively binds to large soluble Aβ protofibrils, was approved in July 2023. Patients receiving lecanemab for 18 months showed a slower progression of disease, with reduced Aβ and better cognition compared with untreated patients (Swanson et al., 2021). The benefits were reported to be greater in men than women (van Dyck et al., 2023) reflecting data obtained in the Thy-Tau22-5xFAD (T5x) mouse model in which the decrease in soluble and insoluble Aβ was greater in brain tissue of male mice compared with females (Davtyan et al., 2019). The efficacy of lecanemab was greater, and the risk of amyloid-related imaging abnormalities (ARIA) was significantly reduced, in *APOE4* non-carriers compared with carriers. However, the data originally presented by van Dyck and colleagues in 2023 were recently reassessed and it was concluded that the impact of lecanemab was less than reported, and was ineffective in females (Kurkinen, 2023).

## Is there evidence of sex-related differences in response to drugs in trial for AD?

In August 2023, 251 clinical trials related to AD and falling under the drug category (clinicaltrials.gov) are in progress or planned (ie described as Active not recruiting, Enrolling by invitation, Not yet recruiting and Recruiting). These include agents for image analysis which are not considered here.

### Drugs that target neurotransmitter pathways

Agents designed to target different neurotransmitters include those acting on the cholinergic system (different formulations of the cholinesterase inhibitors donepezil and huperzine A, the cholinergic antagonist mecamylamine, choline alfoscerate that may increase brain ACh levels and the nicotine patch designed to directly activate nicotinic receptors), the NMDA receptor (memantine and other agents with antagonist action, SAGE-718, AVP-786, AXS-05, huperzine A, Chinese “Smart soup”), the β2-adrenergic receptor agonist CST-2032, and the sigma-2 receptor antagonist, CT1812.

Several agents are being investigated for their effectiveness in controlling AD-related agitation; these include agonists at cannabinoid receptors (cannabidiol, dronabinol and IGC-AD1), α2 adrenergic receptors (gabapentin) and D2 receptors (brexpiprazole), and NMDA receptor antagonists, AXS-05, AVP-786 and “Smart soup”. Agonists and antagonists at different 5-HT receptors (masupirdine, a 5HT-6 receptor antagonist; piromelatine, a 5-HT-1A/1D receptor agonist as well as acting on melatonin MT1/2/3) and the selective serotonin reuptake inhibitor, escitalopram, are also in trial for treating agitation, while trazodone (agonist and antagonist activities at different 5HT receptors), is in trial for AD-related insomnia. Orexin receptor type 2 antagonists (Lemborexant, seltorexant and suvorexant) are also in trial for insomnia and KarXT (M1 receptor agonist) is in trial for the treatment of psychosis.

Clearly, sex-related differences in receptor density or receptor-related signaling opens the possibility that there may be differences in drug efficacy between males and females. Findings, mainly from preclinical studies, suggest that sex hormones influence expression and/or signaling of several receptors (Wilbert-Lampen et al., 2005; Kritzer and Creutz, 2008; Normandin et al., 2015; Bangasser et al., 2016), while sex-related differences in cholinergic activity (Giacobini and Pepeu, 2018) and NMDA receptor function (Wickens et al., 2018; Giacometti and Barker, 2020; Knouse et al., 2022) have been described. There are sex differences in gene expression in noradrenergic receptors (Mulvey et al., 2018) and in circuitry (Sun et al., 2020), and in 5HT transmission, concentration, metabolism and receptor expression (Cosgrove et al., 2007; Sramek et al., 2016; Songtachalert et al., 2018). Analysis of serotonin synthesis by PET using α-[^11^C]methyl-L-tryptophan as a tracer revealed that synthesis was 50% higher in the brains of males compared with females (Nishizawa et al., 1997). Sexual dimorphisms in dopamine receptor expression have also been reported (Williams et al., 2021) with recent metanalysis demonstrating greater binding of the selective D_2_R antagonist [^11^C]raclopride in putamen and caudate of females compared with males (Malén et al., 2022). Sex-related differences in the orexin system (Grafe and Bhatnagar, 2020), and cannabinoid receptors (Rubino and Parolaro, 2011; Normandin et al., 2015) and sigma receptors (Smith et al., 2018), have also been reported. It must be predicted that such differences are likely to significantly affect the efficacy of drugs that target these neurotransmitter receptors or pathways and provide a strong argument in favor of sex subgroup analysis and reporting in clinical trials.

### Agents that target Aβ

Strategies designed to reduce Aβ remain at the heart of discovery in the context of AD. There are several ongoing clinical trials using antibodies that target specific epitopes of Aβ (Song et al., 2022) designed to reduce Aβ burden, including additional trials on the already-licensed aducanumab and lecanemab.

Donanemab, for which a decision from the FDA is expected in 2024, binds to the pyroglutamate-modified, N-terminal form of Aβ that is present in plaques. It retards progression of the disease and decreases Aβ plaques (Mintun et al., 2021; Reardon, 2023a) particularly in those with less tau pathology (Sims et al., 2023). Minimal attention has been paid to assessment of potential sex-related differences in the efficacy of donanemab although one paper suggested that sex did not impact on disease progression rates (Gueorguieva et al., 2023). As in the case of lecanemab, the risk of ARIA was greater in *APOE4* carriers compared with non-carriers.

Among others in trial are solanezumab, which targets the mid-domain of the Aβ peptide and recognizes soluble monomeric Aβ (Doody et al., 2014; Honig et al., 2018), crenezumab, which binds to stable oligomers and inhibits aggregation of Aβ (Ostrowitzki et al., 2022), ganteneumab, which binds to a conformational epitope on Aβ fibrils (Bateman et al., 2022). Data from a phase 2 trial with solanezumab suggested that there may be some sex differences in antibody distribution but this was not further investigated (Farlow et al., 2012). To my knowledge, there are no published data on sex-related differences in distribution, efficacy or pharmacodynamics on any other monoclonal antibody, except lecanemab (see above).

Targeting the N-terminal pyroglutamate Aβ epitope increases microglial-mediated phagocytosis of Aβ (Crehan et al., 2020) and therefore there are an increasing number of antibodies designed to exploit this including ABBV-916 and remternetug, which are currently in phase 2 and 3 trials respectively. Varoglutamstat (PQ912) also reduces production of pyroglutamate Aβ by inhibiting glutaminyl cyclase and is in phase 2 trials. In combination with a mouse pyroglutamate-3 Aβ antibody, it reduced Aβ accumulation and improved cognitive function, in APP/PS1 mice (Hoffmann et al., 2017). Targeting pyroglutamate-modified pE_3_Aβ in the 5XFAD mouse model of AD reduced pyroglutamated and non-pyroglutamated Aβ plaques in male and female mice. Highlighting a sexual dimorphism, soluble Aβ was decreased only in male mice whereas insoluble Aβ was decreased only in female mice (Zagorski et al., 2023). Of course, there is no guarantee that the sex differences identified in mice will translate into humans, but the fact that there are indications of sex-related responses to lecanemab and solanezumab presents a forceful argument for stratification by sex in all clinical trials.

Other approaches to interfere with Aβ accumulation are being investigated, including small molecules designed to prevent oligomer formation, or to block the impact of these oligomers. For instance, ALZ-801, described as anti-oligomer and as an aggregation inhibitor, is currently in phase 2 trials, as is CT1812, a sigma-2 receptor allosteric antagonist, designed to inhibit binding of Aβ oligomers to oligomer receptors thereby reducing Aβ-induced synaptic toxicity. Autoradiographic analysis has shown sex-related differences in sigma 2 receptors binding and suggested that a reduction in receptors is associated specifically with Aβ pathology specifically in female APP/PS1 mice (Sahlholm et al., 2015).

Buntanetap reduces Aβ by inhibiting APP synthesis (Fang et al., 2023) and is currently in phase 2 trials. It improved cognition in APP/PS1 mice and improved synaptic function (Teich et al., 2018) but only female mice were investigated. To date, trials targeting γ-secretase have been unsuccessful (Yiannopoulou et al., 2019) but newer agents have shown some promise (Rynearson et al., 2021). Many trials on BACE1 inhibitors have also been unsuccessful but new classes of drugs that modulate BACE1 activity are in development (Monteiro et al., 2023). It is worth noting that sex differences in γ-secretase and BACE1 have been reported in mice, Increased activity of γ-secretase and increased expression of nicastrin and presenilin have been reported in tissue from 24 month-old female mice (Placanica et al., 2009), while estrogen downregulates BACE1 transcription (Cui et al., 2022). It is also interesting that circulating BACE1 concentrations were significantly higher in a cohort of females compared with males that were at risk of AD (Vergallo et al., 2019).

### Agents that target Tau

Agents in clinical trials aimed at modulating tau are mainly monoclonal antibodies and inhibitors of tau aggregation. Monoclonal antibodies that are currently in trial include Lu AF87908, which recognizes phosphorylated tau (p-tau) protein, APNmAb005, which recognizes a conformational epitope in tau oligomers and JNJ-63733657 and E2814 which recognize the microtubule binding region of tau. The epitope to which E2814 binds, plays a role in seeding and propogation, and is a predominant component of tau tangles (Roberts et al., 2020). Tau-targeting antisense oligonucleotides, NIO752 and BIIB080, that inhibit translation of tau mRNA into the tau protein, and drugs designed to inhibit tau aggregation/self-association LY3372689, TRx0237 and OLX-07010 are also in clinical trial as is nicotinamide riboside, which has been shown to decrease tau phosphorylation (Hou et al., 2018), attenuate cognitive deterioration and improve synaptic plasticity in mouse models of AD (Gong et al., 2013; Hou et al., 2018).

To my knowledge, there are no published reports of sex-related differences in responses to such agents although it is well documented that there are marked differences in tau accumulation in males and females, with greater tau accumulation in brains of female AD patients compared with males (Oveisgharan et al., 2018). A recent study designed to assess tau propagation using regional [^18^F]flortaucipir PET analysis suggested a more widespread increase of tau in female MCI patients compared with males (Shokouhi et al., 2020) and it was shown that naturally-occurring tau antibodies were lower in CSF from female AD patients compared with males (Krestova et al., 2018). Even in cognitively-intact elderly individuals, the age-related increase in p-tau in postmortem brain tissue is enhanced to a greater extent in women than men (Hu et al., 2021). Tau has been coupled with sex-specific genetic variants in men and women that are linked with enhanced risk of AD. SNPs within *DNAJA2*, *FERMT2*, and *TYW5* were associated with tau in women whereas SNPs within *CR1* were associated with Tau in men (Wang X. et al., 2022).

In the absence of clinical data, the findings of preclinical studies that identified sexual dimorphism related to tau are worthy of note. The recombinant protein/tau vaccine AV-1980R, which induces antibodies that inhibit tau aggregation, decreased soluble p-tau in the brains of male and female T5x double transgenic mice but its effect on insoluble p-tau was confined to male mice (Davtyan et al., 2019).

### Drugs to modulate immunity/inflammation

The burgeoning volume of data that implicates perturbation in the immune system and microglial function has triggered a search for agents that modulate both in the hope of altering the course of AD. GWAS has identified polymorphisms in several genes that code for proteins involved in immune function and endow significant risk of AD. One is TREM2 (Guerreiro et al., 2013; Jonsson et al., 2013), which is expressed on microglia and is essential for the clustering of microglia around amyloid plaques (Jay et al., 2017; Lewcock et al., 2020). There is evidence that TREM2 is protective (Zhao et al., 2017; Brown and St George-Hyslop, 2021) as is soluble TREM2, which binds Aβ, prevents its oligomerization and improves cognition in a mouse model of AD (Zhong et al., 2019; Sheng et al., 2021). Consistently, knocking out TREM2 resulted in greater plaque burden; this effect was greater in female mice compared with males (Meilandt et al., 2020). APOE genotype, as well as sex, affects the TREM2-mediated interaction between plaques and microglia. Microglial-plaque interaction correlated with TREM2 expression and was approximately 5 times higher in 5xFAD/APOE3^+/+^ male, compared with female, mice and markedly reduced in 5xFAD/APOE4^+/+^ female mice (Stephen et al., 2019).

Clinical trials on AL002, an antibody targeting TREM2, are ongoing and a second antibody, DNL919, has been placed on clinical hold while further assessment of its safety/toxicity is investigated. These trials make no specific reference to analysis of sex-related differences though this is clearly relevant given the available preclinical data.

Other drugs in clinical trials that are designed to modulate microglia include sodium oligomannate (GV-971), mastinib and the CSF1R antagonist JNJ-40346527. Another CSF1R antagonist, PLX3397, has been assessed in a clinical trial but the EU Clinical Trials Register reports that the trial was ended prematurely. However because its action is similar to that of JNJ-40346527, it is relevant to consider the data which indicate that PLX3397 exerts sex-dependent effects. It depleted microglia to a greater extent in female, compared with male, rats (Sharon et al., 2022) and, in Tg2541 mice, it decreased tau, and extended the life span of female, but not male mice (Johnson et al., 2023). It was further shown that plasma and brain PLX3397 levels were increased to a greater extent in male, compared with female, mice indicating that sex impacts on PLX3397 pharmacokinetics. This suggests that PLX3397 treatment should be tailored to sex to ensure benefit in males and females, in mice in the first instance.

Masitinib modulates microglia probably through inhibition of the M-CSF receptor kinase 1, although it also inhibits the tyrosine kinases c-kit, fyn and lyn, and PDGF and FGF receptors. Analysis in cancer patients has identified sex-related differences in pharmacokinetics and, in particular, slower elimination of tyrosine kinase inhibitors in females (Huang et al., 2022; Özdemir et al., 2022). Other inhibitors of tyrosine kinases that are also in clinical trial for the treatment of AD include dasatinib, a potent inhibitor of Abl and Src and the JAK inhibitor, baricitinib (Matsushita et al., 2023), although sex-related differences in the pharmacokinetics of these have not been reported.

Semaphorin 4D and galactin 3 redirect microglia toward an inflammatory phenotype (Smith et al., 2015; Tan et al., 2021) and are upregulated in AD (Tan et al., 2021; Evans et al., 2022); antibodies directed at both are currently in Phase 1 and/or 2 trials. There is some evidence of a sexual dimorphic effect of galactin 3 in mouse models of stroke (Mijailović et al., 2022). Daratumumab, a CD38 antibody is also being assessed as a potential disease-modifying strategy in AD since CD38 also modulates microglial activation and has been shown to enhance Aβ accumulation (Blacher et al., 2015).

Mutations in the gene encoding sortilin1 have been linked with increased risk of AD and an anti-sortilin 1 antibody, AL-001, is currently in clinical trials for AD. One mutation, SORL1 SNP 4 (rs661057), is associated with an increased risk of AD in women (Cellini et al., 2009).

Other agents in clinical trials designed to impact on microglia and reduce neuroinflammation include those that aim to reduce inflammatory cytokines, or the impact of these cytokines, like lenalidomide (Valera et al., 2015), L-serine (Ye et al., 2021) and XPro1595 (MacPherson et al., 2017) and, at least in the case of XPro1595, its modulatory effects on stress-induced inflammatory changes were shown to be sex-dependent (Eidson et al., 2019). The antiretroviral agent, lamivudine, which improves age-related cognitive function and modulates genes related to the Type 1 interferon response (Li et al., 2021), is currently in early phase trials and is known to exert sex-related effects (Anderson et al., 2003). The cannabinoids, SCI-100 and palmitoylethanolamide (Landucci et al., 2022), are also in clinical trial because of their reported anti-inflammatory action and, when assessed for the treatment of neuropathic pain, responses to palmitoylethanolamide were shown to be sex-dependent (Morera et al., 2015).

It is abundantly clear that microglial phenotype and function are markedly affected by sex with an ever-growing literature suggesting a disproportionate impact of microglial activation and inflammation in females in the context of AD (Kodama et al., 2020; Casaletto et al., 2022; Lynch, 2022). This means that particular attention should be paid to sex when agents that affect their function are in clinical use and demands that clinical trials should be designed with specific emphasis placed on specifically assessing sex-related responses to drugs.

As indicated in the paragraphs above, a significant part of the work investigating the role of inflammation in AD has been derived from animal studies. While many of these suggest that inflammation drives amyloid accumulation, there are contradictory data, perhaps arising from differences between the different models of AD, as pointed out in a recent review (Xie et al., 2021). Furthermore, it is without doubt that amyloid contributes to inflammatory changes (Wang S. et al., 2022) and therefore further study is necessary to clarify the temporal relationship between inflammation and amyloid accumulation.

Studies using positron emission tomography (PET) have reported that microglial activation, as assessed by ^11^C-PBR28, correlates with accumulation of amyloid (^18^F-flutemetamol) more so in MCI, compared with tau (^18^F-AV1451) where the correlation is stronger in established AD (Dani et al., 2018). This indicates that an important factor in consideration of treatments that target microglial activation/inflammation is timing, which was emphasized by others who reported a “biphasic trajectory of inflammation” where the correlation between inflammation and amyloid accumulation varied with the stage of disease (Ismail et al., 2020).

### Drugs targeting oxidative stress

Oxidative stress is a feature of AD and homocysteine concentration, which is a marker for oxidative stress, was greater in men with AD compared with women (Tenkorang et al., 2018). This is significant because a number of agents that target oxidative stress are currently in trial for AD including n-3 fatty acids, DHA and EPA, the fatty acid synthase inhibitor, CMS121, the free radical scavenger, hydralazaine hydrochloride and Flos gossypii flavonoids. Sex-related differences in responses to n-3 fatty acids have been reported in mice with DHA reducing social isolation-induced anxiety and depressive-like behaviors in male mice but not in female mice (Davis et al., 2017). In human studies, a strong positive correlation between dietary n-3 fatty acids and cognition was observed in females but not males (Lassek and Gaulin, 2011) while dietary supplementation with n-3 fatty acids improved episodic memory in females but not males (Stonehouse et al., 2013). Hydralazine hydrochloride eexerts its antioxidative effects by activating the Nrf2 pathway and the evidence indicates that sex is a variable in Nrf2 activation. Specifically, dimethylfumarate (DMF), which increases Nrf2 activation, exerted an effect in microglia prepared from the cortex of female mice but not male mice (Mela et al., 2022).

### Drugs targeting metabolism

Disruption of metabolic processes are well-described features of AD and these include glucose hypometabolism (Hammond et al., 2020; Hammond and Lin, 2022) and a shift from glucose to lipid metabolism (Demarest et al., 2020). A recent analysis revealed that brain metabolism decreased over a 2 year period in women with AD, as assessed by ^18^F fluorodeoxyglucose-PET, and that this was correlated with circulating Aβ42:Aβ40. Further transcriptomic analysis identified that the number of differentially expressed genes, particularly those related to glucose metabolism, was 3 times greater in female, compared with male AD, patients (Park et al., 2023). Similar sex-specific differences have been reported in animal models of AD (Guillot-Sestier et al., 2021; Strefeler et al., 2023). Impaired mitochondrial function is also a recognized change in the brains of AD patients (Wang et al., 2020) and animal models of AD (Fang et al., 2019; Demarest et al., 2020) and the evidence indicates that mitochondrial metabolism is particularly compromised in female APP/PS1 mice (Demarest et al., 2020; O’Neill et al., 2022).

Unsurprisingly, a number of drugs that aim to restore metabolism are in development, and a few are in clinical trials including nicotinamide riboside and dapaglifozin, which are designed to improve mitochondrial function and reduce blood glucose by inhibiting the sodium-glucose co-transporter-2 (SGLT2). There is some evidence suggesting that SGLT2 may exert sex-specific effects (Rivera et al., 2023).

The medium chain triglyceride, tricaprilin, is also in clinical trial. It is ultimately metabolized to ketones which appear to exert beneficial effects in AD (Jensen et al., 2020) with evidence from preclinical studies indicating that ketone supplementation decreased β-hydroxybutyrate and increased blood glucose in older female rats and had the opposite effect in male rats, while body weight increased to a greater extent in males (Kovács et al., 2020). Interestingly, in TBI, the protective effect of β-hydroxybutyrate appears to be confined to males (Greco et al., 2020).

Type 2 diabetes confers a significant risk of developing AD and a recent study that analyzed data from 450,000 individuals determined that females with type 2 diabetes had a higher risk of developing AD than men with type 2 diabetes (Zhou et al., 2023). Significantly, the evidence suggests that type 2 diabetes is more prevalent in women than men (Ashraf et al., 2021). Type 2 diabetes and AD share similarities, particularly insulin resistance (Arnold et al., 2018). These similarities and the evidence from animal studies demonstrating that intranasal insulin improved cognition in APP/PS1 mice (Mao et al., 2016) and also in 3xTg AD mice, aged animals and Aβ-treated animals (Ohyagi et al., 2019), triggered an interest in assessing insulin as a potential therapy in AD. Intranasal insulin was reported to exert no significant effect on cognition in a recent study in which data from males and females were combined (Craft et al., 2020). However an earlier study reported sex-dependent effects in MCI and AD patients; improved cognitive function was observed in males but not females and the effect was greater in ApoE ε4-negative males, but not females (Claxton et al., 2013). Further studies are clearly required to gain clarity on effects and there are currently 2 trials in progress.

Other agents designed to improve insulin sensitivity and signaling are also in clinical trial, including semaglutide, a glucagon-like peptide-1 (GLP-1) agonist that enhances insulin signaling in the brain. A recent analysis of 3 large trials concluded that treatment of patients with type 2 diabetes with semaglutide or liraglutide reduced the incidence of dementia (Nørgaard et al., 2022). Although similar results were reported in males and females, sex-related differences in the effects of GLP-1 agonists have been observed; for example, weight loss, a greater decrease in glycated hemoglobin and a greater improvement in β cell function were all recorded in females (Rentzeperi et al., 2022). GLP-1 agonists were also more effective in reducing cardiovascular events in females with type 2 diabetes compared with males (Raparelli et al., 2020).

Metformin is currently in trial as a potential disease modifying agent in AD. A study in the PDAPP (J9) mouse model (AβPP mice) of AD revealed that cognitive function improved in metformin-treated, compared with control-treated female AβPP mice whereas it deteriorated in males (DiTacchio et al., 2015). There are no reports on sex-related differences in response to metformin in AD patients as highlighted recently (Chaudhari et al., 2020).

## Conclusion

Until relatively recently, recruitment for clinical trials did not give due consideration to the possible impact of sex on outcomes and reports often did not stratify findings by sex. In the context of AD, recruitment now commonly reflects the greater prevalence of the disease in women. However reporting of sex-specific responses to drugs remains an issue. Indeed a recent metanalysis found that only 7 studies out of the 56 assessed, specifically evaluated and reported on the impact of sex (Martinkova et al., 2021) although it has been observed that some data are publicly available on the US FDA website, Drugs@FDA (Schwartz and Weintraub, 2021).

In this review, drugs in clinical trial for AD have been grouped into those targeting neurotransmitters, Aβ, tau, immunity/inflammation, oxidative stress and metabolism. For each of these targets, there is reason to predict sex differences in responses to drugs, based on evidence mainly from preclinical analysis. However this speculation requires rigorous assessment and emphasizes the importance of evaluating sex as a variable. There is a strong argument favoring a move toward including analysis of changes by sex in all clinical trials and reporting the results of trials in a sex-stratified manner.

## Author contributions

ML: Writing – original draft, Writing – review and editing.

## References

[B1] Anderson P. L. Kakuda T. N. Kawle S. Fletcher C. V. (2003). Antiviral dynamics and sex differences of zidovudine and lamivudine triphosphate concentrations in HIV-infected individuals. *AIDS* 17 2159–2168. 10.1097/00002030-200310170-00003 14523272

[B2] Arnold S. E. Arvanitakis Z. Macauley-Rambach S. L. Koenig A. M. Wang H. Y. Ahima R. S. (2018). Brain insulin resistance in type 2 diabetes and Alzheimer disease: concepts and conundrums. *Nat. Rev. Neurol.* 14 168–181. 10.1038/nrneurol.2017.185 29377010 PMC6098968

[B3] Ashraf G. M. Ebada M. A. Suhail M. Ali A. Uddin M. S. Bilgrami A. L. (2021). Dissecting sex-related cognition between Alzheimer’s disease and diabetes: from molecular mechanisms to potential therapeutic strategies. *Oxid. Med. Cell. Longev.* 2021:4572471. 10.1155/2021/4572471 33747345 PMC7960032

[B4] Athnaiel O. Cantillo S. Paredes S. Knezevic N. N. (2023). The role of sex hormones in pain-related conditions. *Int. J. Mol. Sci.* 24:1866. 10.3390/ijms24031866 PMC9915903 36768188

[B5] Bangasser D. A. Wiersielis K. R. Khantsis S. (2016). Sex differences in the locus coeruleus-norepinephrine system and its regulation by stress. *Brain Res.* 1641 177–188. 10.1016/j.brainres.2015.11.021 26607253 PMC4875880

[B6] Barha C. K. Liu-Ambrose T. (2018). Exercise and the aging brain: considerations for sex differences. *Brain Plast.* 4 53–63. 10.3233/BPL-180067 30564546 PMC6296261

[B7] Barha C. K. Davis J. C. Falck R. S. Nagamatsu L. S. Liu-Ambrose T. (2017). Sex differences in exercise efficacy to improve cognition: a systematic review and meta-analysis of randomized controlled trials in older humans. *Front. Neuroendocrinol.* 46:71–85. 10.1016/j.yfrne.2017.04.002 28442274

[B8] Bateman R. J. Cummings J. Schobel S. Salloway S. Vellas B. Boada M. (2022). Gantenerumab: an anti-amyloid monoclonal antibody with potential disease-modifying effects in early Alzheimer’s disease. *Alzheimers Res. Ther.* 14:178. 10.1186/s13195-022-01110-8 36447240 PMC9707418

[B9] Bellenguez C. Küçükali F. Jansen I. E. Kleineidam L. Moreno-Grau S. Amin N. (2022). New insights into the genetic etiology of Alzheimer’s disease and related dementias. *Nat. Genet.* 54 412–436. 10.1038/s41588-022-01024-z 35379992 PMC9005347

[B10] Bennett C. Green J. Ciancio M. Goral J. Pitstick L. Pytynia M. (2022). Dietary folic acid deficiency impacts hippocampal morphology and cortical acetylcholine metabolism in adult male and female mice. *Nutr. Neurosci.* 25 2057–2065. 10.1080/1028415X.2021.1932242 34042561

[B11] Blacher E. Dadali T. Bespalko A. Haupenthal V. J. Grimm M. O. Hartmann T. (2015). Alzheimer’s disease pathology is attenuated in a CD38-deficient mouse model. *Ann. Neurol.* 78 88–103. 10.1002/ana.24425 25893674 PMC6929692

[B12] Bloomberg M. Dugravot A. Dumurgier J. Kivimaki M. Fayosse A. Steptoe A. (2021). Sex differences and the role of education in cognitive ageing: analysis of two UK-based prospective cohort studies. *Lancet Public Health* 6 e106–e115. 10.1016/S2468-2667(20)30258-9 33516287 PMC8141610

[B13] Brooks L. R. K. Mias G. I. (2019). Data-driven analysis of age, sex, and tissue effects on gene expression variability in Alzheimer’s disease. *Front. Neurosci.* 13:392. 10.3389/fnins.2019.00392 31068785 PMC6491842

[B14] Brown G. C. St George-Hyslop P. (2021). Does soluble TREM2 protect against Alzheimer’s disease? *Front. Aging Neurosci.* 13:834697. 10.3389/fnagi.2021.834697 35153729 PMC8831327

[B15] Buccafusco J. J. Jackson W. J. Stone J. D. Terry A. V. (2003). Sex dimorphisms in the cognitive-enhancing action of the Alzheimer’s drug donepezil in aged rhesus monkeys. *Neuropharmacology* 44 381–389. 10.1016/s0028-3908(02)00378-7 12604096

[B16] Buckley R. F. Mormino E. C. Amariglio R. E. Properzi M. J. Rabin J. S. Lim Y. Y. (2018). Sex, amyloid, and APOE epsilon4 and risk of cognitive decline in preclinical Alzheimer’s disease: findings from three well-characterized cohorts. *Alzheimers Dement.* 14 1193–1203. 10.1016/j.jalz.2018.04.010 29803541 PMC6131023

[B17] Budd Haeberlein S. Aisen P. S. Barkhof F. Chalkias S. Chen T. Cohen S. (2022). Two randomized Phase 3 studies of aducanumab in early Alzheimer’s disease. *J. Prev. Alzheimers Dis.* 9 197–210. 10.14283/jpad.2022.30 35542991

[B18] Burkhardt M. S. Foster J. K. Laws S. M. Baker L. D. Craft S. Gandy S. E. (2004). Oestrogen replacement therapy may improve memory functioning in the absence of APOE epsilon4. *J. Alzheimers Dis.* 6 221–228. 10.3233/jad-2004-6302 15201477

[B19] Butterfield D. A. Halliwell B. (2019). Oxidative stress, dysfunctional glucose metabolism and Alzheimer disease. *Nat. Rev. Neurosci.* 20 148–160. 10.1038/s41583-019-0132-6 30737462 PMC9382875

[B20] Cáceres A. González J. R. (2020). Female-specific risk of Alzheimer’s disease is associated with tau phosphorylation processes: a transcriptome-wide interaction analysis. *Neurobiol. Aging* 96 104–108. 10.1016/j.neurobiolaging.2020.08.020 32977080

[B21] Canevelli M. Quarata F. Remiddi F. Lucchini F. Lacorte E. Vanacore N. (2017). Sex and gender differences in the treatment of Alzheimer’s disease: a systematic review of randomized controlled trials. *Pharmacol. Res.* 115 218–223. 10.1016/j.phrs.2016.11.035 27913252

[B22] Casaletto K. B. Nichols E. Aslanyan V. Simone S. M. Rabin J. S. La Joie R. (2022). Sex-specific effects of microglial activation on Alzheimer’s disease proteinopathy in older adults. *Brain* 145 3536–3545. 10.1093/brain/awac257 35869598 PMC10233295

[B23] Cellini E. Tedde A. Bagnoli S. Pradella S. Piacentini S. Sorbi S. (2009). Implication of sex and SORL1 variants in Italian patients with Alzheimer disease. *Arch. Neurol.* 66 1260–1266. 10.1001/archneurol.2009.101 19822782

[B24] Chaudhari K. Reynolds C. D. Yang S. H. (2020). Metformin and cognition from the perspectives of sex, age, and disease. *Geroscience* 42 97–116. 10.1007/s11357-019-00146-3 31897861 PMC7031469

[B25] Choi S. H. Kim S. Y. Na H. R. Kim B. K. Yang D. W. Kwon J. C. (2008). Effect of ApoE genotype on response to donepezil in patients with Alzheimer’s disease. *Dement. Geriatr. Cogn. Disord.* 25 445–450. 10.1159/000124752 18401173

[B26] Claxton A. Baker L. D. Wilkinson C. W. Trittschuh E. H. Chapman D. Watson G. S. (2013). Sex and ApoE genotype differences in treatment response to two doses of intranasal insulin in adults with mild cognitive impairment or Alzheimer’s disease. *J. Alzheimers Dis.* 35 789–797. 10.3233/JAD-122308 23507773 PMC4144993

[B27] Coales I. Tsartsalis S. Fancy N. Weinert M. Clode D. Owen D. (2022). Alzheimer’s disease-related transcriptional sex differences in myeloid cells. *J. Neuroinflammation* 19:247. 10.1186/s12974-022-02604-w 36199077 PMC9535846

[B28] Contreras J. A. Aslanyan V. Albrecht D. S. Mack W. J. Pa J. Alzheimer’s Disease Neuroimaging Initiative (ADNI) (2022). Higher baseline levels of CSF inflammation increase risk of incident mild cognitive impairment and Alzheimer’s disease dementia. *Alzheimers Dement.* 14:e12346. 10.1002/dad2.12346 36187197 PMC9484791

[B29] Corder E. H. Saunders A. M. Strittmatter W. J. Schmechel D. E. Gaskell P. C. Small G. W. (1993). Gene dose of apolipoprotein E type 4 allele and the risk of Alzheimer’s disease in late onset families. *Science* 261 921–923. 10.1126/science.8346443 8346443

[B30] Cosgrove K. P. Mazure C. M. Staley J. K. (2007). Evolving knowledge of sex differences in brain structure, function, and chemistry. *Biol. Psychiatry* 62 847–855. 10.1016/j.biopsych.2007.03.001 17544382 PMC2711771

[B31] Craft S. Raman R. Chow T. W. Rafii M. S. Sun C. K. Rissman R. A. (2020). Safety, efficacy, and feasibility of intranasal insulin for the treatment of mild cognitive impairment and Alzheimer disease dementia: a randomized clinical trial. *JAMA Neurol.* 77 1099–1109. 10.1001/jamaneurol.2020.1840 32568367 PMC7309571

[B32] Crawford F. Abdullah L. Schinka J. Suo Z. Gold M. Duara R. (2000). Gender-specific association of the angiotensin converting enzyme gene with Alzheimer’s disease. *Neurosci. Lett.* 280 215–219. 10.1016/s0304-3940(00)00791-6 10675799

[B33] Crehan H. Liu B. Kleinschmidt M. Rahfeld J. U. Le K. X. Caldarone B. J. (2020). Effector function of anti-pyroglutamate-3 Abeta antibodies affects cognitive benefit, glial activation and amyloid clearance in Alzheimer’s-like mice. *Alzheimers Res. Ther.* 12:12. 10.1186/s13195-019-0579-8 31931873 PMC6958628

[B34] Cui J. Ait-Ghezala G. Sambamurti K. Gao F. Shen Y. Li R. (2022). Sex-specific regulation of beta-secretase: a novel estrogen response element (ERE)-dependent mechanism in Alzheimer’s disease. *J. Neurosci.* 42 1154–1165. 10.1523/JNEUROSCI.0864-20.2021 34903570 PMC8824503

[B35] Dani M. Wood M. Mizoguchi R. Fan Z. Walker Z. Morgan R. (2018). Microglial activation correlates in vivo with both tau and amyloid in Alzheimer’s disease. *Brain* 141 2740–2754. 10.1093/brain/awy188 30052812

[B36] Davis D. J. Hecht P. M. Jasarevic E. Beversdorf D. Q. Will M. J. Fritsche K. (2017). Sex-specific effects of docosahexaenoic acid (DHA) on the microbiome and behavior of socially isolated mice. *Brain Behav. Immun.* 59 38–48. 10.1016/j.bbi.2016.09.003 27621225

[B37] Davis M. L. Barrett A. M. (2009). Selective benefit of donepezil on oral naming in Alzheimer’s disease in men compared to women. *CNS Spectr.* 14 175–176. 10.1017/s1092852900020174 19407728 PMC2928142

[B38] Davtyan H. Hovakimyan A. Kiani Shabestari S. Antonyan T. Coburn M. A. Zagorski K. (2019). Testing a MultiTEP-based combination vaccine to reduce Abeta and tau pathology in Tau22/5xFAD bigenic mice. *Alzheimers Res. Ther.* 11:107. 10.1186/s13195-019-0556-2 31847886 PMC6918571

[B39] Demarest T. G. Varma V. R. Estrada D. Babbar M. Basu S. Mahajan U. V. (2020). Biological sex and DNA repair deficiency drive Alzheimer’s disease via systemic metabolic remodeling and brain mitochondrial dysfunction. *Acta Neuropathol.* 140 25–47. 10.1007/s00401-020-02152-8 32333098 PMC7537767

[B40] Demetrius L. A. Eckert A. Grimm A. (2021). Sex differences in Alzheimer’s disease: metabolic reprogramming and therapeutic intervention. *Trends Endocrinol. Metab.* 32 963–979. 10.1016/j.tem.2021.09.004 34654630

[B41] Deming Y. Dumitrescu L. Barnes L. L. Thambisetty M. Kunkle B. Gifford K. A. (2018). Sex-specific genetic predictors of Alzheimer’s disease biomarkers. *Acta Neuropathol.* 136 857–872. 10.1007/s00401-018-1881-4 29967939 PMC6280657

[B42] Deng Y. T. Li Y. Z. Huang S. Y. Ou Y. N. Zhang W. Chen S. D. (2022). Association of life course adiposity with risk of incident dementia: a prospective cohort study of 322,336 participants. *Mol. Psychiatry* 27 3385–3395. 10.1038/s41380-022-01604-9 35538193

[B43] DiTacchio K. A. Heinemann S. F. Dziewczapolski G. (2015). Metformin treatment alters memory function in a mouse model of Alzheimer’s disease. *J. Alzheimers Dis.* 44 43–48. 10.3233/JAD-141332 25190626 PMC9057391

[B44] Dong C. Zhou C. Fu C. Hao W. Ozaki A. Shrestha N. (2022). Sex differences in the association between cardiovascular diseases and dementia subtypes: a prospective analysis of 464,616 UK Biobank participants. *Biol. Sex Differ.* 13:21. 10.1186/s13293-022-00431-5 35526028 PMC9080133

[B45] Doody R. S. Thomas R. G. Farlow M. Iwatsubo T. Vellas B. Joffe S. (2014). Phase 3 trials of solanezumab for mild-to-moderate Alzheimer’s disease. *N. Engl. J. Med.* 370 311–321. 10.1056/NEJMoa1312889 24450890

[B46] Edwards L. La Joie R. Iaccarino L. Strom A. Baker S. L. Casaletto K. B. (2021). Multimodal neuroimaging of sex differences in cognitively impaired patients on the Alzheimer’s continuum: greater tau-PET retention in females. *Neurobiol. Aging* 105 86–98. 10.1016/j.neurobiolaging.2021.04.003 34049062 PMC8820163

[B47] Eidson L. N. deSousa Rodrigues M. E. Johnson M. A. Barnum C. J. Duke B. J. Yang Y. (2019). Chronic psychological stress during adolescence induces sex-dependent adulthood inflammation, increased adiposity, and abnormal behaviors that are ameliorated by selective inhibition of soluble tumor necrosis factor with XPro1595. *Brain Behav. Immun.* 81 305–316. 10.1016/j.bbi.2019.06.027 31251975 PMC8597195

[B48] Evans E. E. Mishra V. Mallow C. Gersz E. M. Balch L. Howell A. (2022). Semaphorin 4D is upregulated in neurons of diseased brains and triggers astrocyte reactivity. *J. Neuroinflammation* 19:200. 10.1186/s12974-022-02509-8 35933420 PMC9356477

[B49] Fan C. C. Banks S. J. Thompson W. K. Chen C. H. McEvoy L. K. Tan C. H. (2020). Sex-dependent autosomal effects on clinical progression of Alzheimer’s disease. *Brain* 143 2272–2280. 10.1093/brain/awaa164 32591829 PMC7364740

[B50] Fang C. Hernandez P. Liow K. Damiano E. Zetterberg H. Blennow K. (2023). Buntanetap, a novel translational inhibitor of multiple neurotoxic proteins, proves to be safe and promising in both Alzheimer’s and Parkinson’s patients. *J. Prev. Alzheimers Dis.* 10 25–33. 10.14283/jpad.2022.84 36641607

[B51] Fang E. F. Hou Y. Palikaras K. Adriaanse B. A. Kerr J. S. Yang B. (2019). Mitophagy inhibits amyloid-beta and tau pathology and reverses cognitive deficits in models of Alzheimer’s disease. *Nat. Neurosci.* 22 401–412. 10.1038/s41593-018-0332-9 30742114 PMC6693625

[B52] Farlow M. R. Lahiri D. K. Poirier J. Davignon J. Schneider L. Hui S. L. (1998). Treatment outcome of tacrine therapy depends on apolipoprotein genotype and gender of the subjects with Alzheimer’s disease. *Neurology* 50 669–677. 10.1212/wnl.50.3.669 9521254

[B53] Farlow M. Arnold S. E. van Dyck C. H. Aisen P. S. Snider B. J. Porsteinsson A. P. (2012). Safety and biomarker effects of solanezumab in patients with Alzheimer’s disease. *Alzheimers Dement.* 8 261–271. 10.1016/j.jalz.2011.09.224 22672770

[B54] Fehér Á. Juhász A. Pákáski M. Kálmán J. Janka Z. (2015). Genetic analysis of the RELN gene: gender specific association with Alzheimer’s disease. *Psychiatry Res.* 230 716–718. 10.1016/j.psychres.2015.09.021 26384575

[B55] Ferris S. Lane R. Sfikas N. Winblad B. Farlow M. Feldman H. H. (2009). Effects of gender on response to treatment with rivastigmine in mild cognitive impairment: a post hoc statistical modeling approach. *Gend. Med.* 6 345–355. 10.1016/j.genm.2009.06.004 19682661

[B56] Giacobini E. Pepeu G. (2018). Sex and gender differences in the brain cholinergic system and in the response to therapy of Alzheimer disease with cholinesterase inhibitors. *Curr. Alzheimer Res.* 15 1077–1084. 10.2174/1567205015666180613111504 29895246

[B57] Giacometti L. L. Barker J. M. (2020). Sex differences in the glutamate system: implications for addiction. *Neurosci. Biobehav. Rev.* 113 157–168. 10.1016/j.neubiorev.2020.03.010 32173404 PMC7225077

[B58] Gong B. Pan Y. Vempati P. Zhao W. Knable L. Ho L. (2013). Nicotinamide riboside restores cognition through an upregulation of proliferator-activated receptor-gamma coactivator 1alpha regulated beta-secretase 1 degradation and mitochondrial gene expression in Alzheimer’s mouse models. *Neurobiol. Aging* 34 1581–1588. 10.1016/j.neurobiolaging.2012.12.005 23312803 PMC3632303

[B59] Gong J. Harris K. Lipnicki D. M. Castro-Costa E. Lima-Costa M. F. Diniz B. S. (2023). Sex differences in dementia risk and risk factors: individual-participant data analysis using 21 cohorts across six continents from the COSMIC consortium. *Alzheimers Dement.* 19 3365–3378. 10.1002/alz.12962 36790027 PMC10955774

[B60] Grafe L. A. Bhatnagar S. (2020). The contribution of orexins to sex differences in the stress response. *Brain Res.* 1731:145893. 10.1016/j.brainres.2018.07.026 30081036 PMC6360123

[B61] Greco T. Vespa P. M. Prins M. L. (2020). Alternative substrate metabolism depends on cerebral metabolic state following traumatic brain injury. *Exp. Neurol.* 329:113289. 10.1016/j.expneurol.2020.113289 32247790 PMC8168752

[B62] Gregory S. Ritchie C. W. Ritchie K. Shannon O. Stevenson E. J. Muniz-Terrera G. (2022). Mediterranean diet score is associated with greater allocentric processing in the EPAD LCS cohort: a comparative analysis by biogeographical region. *Front. Aging* 3:1012598. 10.3389/fragi.2022.1012598 36568511 PMC9780498

[B63] Gueorguieva I. Willis B. A. Chua L. Chow K. Ernest C. S. Wang J. (2023). Donanemab exposure and efficacy relationship using modeling in Alzheimer’s disease. *Alzheimers Dement.* 9:e12404. 10.1002/trc2.12404 37388759 PMC10301702

[B64] Guerreiro R. Wojtas A. Bras J. Carrasquillo M. Rogaeva E. Majounie E. (2013). TREM2 variants in Alzheimer’s disease. *N. Engl. J. Med.* 368 117–127. 10.1056/NEJMoa1211851 23150934 PMC3631573

[B65] Guillot-Sestier M. V. Araiz A. R. Mela V. Gaban A. S. O’Neill E. Joshi L. (2021). Microglial metabolism is a pivotal factor in sexual dimorphism in Alzheimer’s disease. *Commun. Biol.* 4:711. 10.1038/s42003-021-02259-y 34112929 PMC8192523

[B66] Guo L. Zhong M. B. Zhang L. Zhang B. Cai D. (2022). Sex differences in Alzheimer’s disease: insights from the multiomics landscape. *Biol. Psychiatry* 91 61–71. 10.1016/j.biopsych.2021.02.968 33896621 PMC8996342

[B67] Hammond T. C. Lin A. L. (2022). Glucose metabolism is a better marker for predicting clinical Alzheimer’s disease than amyloid or tau. *J. Cell. Immunol.* 4 15–18. 35373192 PMC8975178

[B68] Hammond T. C. Xing X. Wang C. Ma D. Nho K. Crane P. K. (2020). Beta-amyloid and tau drive early Alzheimer’s disease decline while glucose hypometabolism drives late decline. *Commun. Biol.* 3:352. 10.1038/s42003-020-1079-x 32632135 PMC7338410

[B69] Haywood W. M. Mukaetova-Ladinska E. B. (2006). Sex influences on cholinesterase inhibitor treatment in elderly individuals with Alzheimer’s disease. *Am. J. Geriatr. Pharmacother.* 4 273–286. 10.1016/j.amjopharm.2006.09.009 17062329

[B70] Hoffmann T. Meyer A. Heiser U. Kurat S. Böhme L. Kleinschmidt M. (2017). Glutaminyl cyclase inhibitor PQ912 improves cognition in mouse models of Alzheimer’s disease-studies on relation to effective target occupancy. *J. Pharmacol. Exp. Ther.* 362 119–130. 10.1124/jpet.117.240614 28446518

[B71] Honig L. S. Vellas B. Woodward M. Boada M. Bullock R. Borrie M. (2018). Trial of solanezumab for mild dementia due to Alzheimer’s disease. *N. Engl. J. Med.* 378 321–330. 10.1056/NEJMoa1705971 29365294

[B72] Hou X. Adeosun S. O. Zhang Q. Barlow B. Brents M. Zheng B. (2015). Differential contributions of ApoE4 and female sex to BACE1 activity and expression mediate Abeta deposition and learning and memory in mouse models of Alzheimer’s disease. *Front. Aging Neurosci.* 7:207. 10.3389/fnagi.2015.00207 26582141 PMC4628114

[B73] Hou Y. Lautrup S. Cordonnier S. Wang Y. Croteau D. L. Zavala E. (2018). NAD(+) supplementation normalizes key Alzheimer’s features and DNA damage responses in a new AD mouse model with introduced DNA repair deficiency. *Proc. Natl. Acad. Sci. U.S.A.* 115 E1876–E1885. 10.1073/pnas.1718819115 29432159 PMC5828618

[B74] Hu Y. T. Boonstra J. McGurran H. Stormmesand J. Sluiter A. Balesar R. (2021). Sex differences in the neuropathological hallmarks of Alzheimer’s disease: focus on cognitively intact elderly individuals. *Neuropathol. Appl. Neurobiol.* 47 958–966. 10.1111/nan.12729 33969531 PMC9290663

[B75] Huang B. Cao G. Duan Y. Yan S. Yan M. Yin P. (2020). Gender differences in the association between hearing loss and cognitive function. *Am. J. Alzheimers Dis. Other Dement.* 35:1533317519871167. 10.1177/1533317519871167 31510756 PMC10623990

[B76] Huang Y. Cho H. J. Stranger B. E. Huang R. S. (2022). Sex dimorphism in response to targeted therapy and immunotherapy in non-small cell lung cancer patients: a narrative review. *Transl. Lung Cancer Res.* 11 920–934. 10.21037/tlcr-21-1013 35693273 PMC9186178

[B77] Ihira H. Sawada N. Inoue M. Yasuda N. Yamagishi K. Charvat H. (2022). Association between physical activity and risk of disabling dementia in Japan. *JAMA Netw. Open* 5:e224590. 10.1001/jamanetworkopen.2022.4590 35348711 PMC8965633

[B78] Ismail R. Parbo P. Madsen L. S. Hansen A. K. Hansen K. V. Schaldemose J. L. (2020). The relationships between neuroinflammation, beta-amyloid and tau deposition in Alzheimer’s disease: a longitudinal PET study. *J. Neuroinflammation* 17:151. 10.1186/s12974-020-01820-6 32375809 PMC7203856

[B79] Jay T. R. von Saucken V. E. Landreth G. E. (2017). TREM2 in neurodegenerative diseases. *Mol. Neurodegener.* 12:56. 10.1186/s13024-017-0197-5 28768545 PMC5541421

[B80] Jensen N. J. Wodschow H. Z. Nilsson M. Rungby J. (2020). Effects of ketone bodies on brain metabolism and function in neurodegenerative diseases. *Int. J. Mol. Sci.* 21:8767. 10.3390/ijms21228767 33233502 PMC7699472

[B81] Johnson N. R. Yuan P. Castillo E. Lopez T. P. Yue W. Bond A. (2023). CSF1R inhibitors induce a sex-specific resilient microglial phenotype and functional rescue in a tauopathy mouse model. *Nat. Commun.* 14:118. 10.1038/s41467-022-35753-w 36624100 PMC9829908

[B82] Jonsson T. Stefansson H. Steinberg S. Jonsdottir I. Jonsson P. V. Snaedal J. (2013). Variant of TREM2 associated with the risk of Alzheimer’s disease. *N. Engl. J. Med.* 368 107–116. 10.1056/NEJMoa1211103 23150908 PMC3677583

[B83] Kapogiannis D. Mattson M. P. (2011). Disrupted energy metabolism and neuronal circuit dysfunction in cognitive impairment and Alzheimer’s disease. *Lancet Neurol.* 10 187–198. 10.1016/S1474-4422(10)70277-5 21147038 PMC3026092

[B84] Khera A. Vega G. L. Das S. R. Ayers C. McGuire D. K. Grundy S. M. (2009). Sex differences in the relationship between C-reactive protein and body fat. *J. Clin. Endocrinol. Metab.* 94 3251–3258. 10.1210/jc.2008-2406 19567538 PMC2741708

[B85] Kilian C. Klinger S. Rehm J. Manthey J. (2023). Alcohol use, dementia risk, and sex: a systematic review and assessment of alcohol-attributable dementia cases in Europe. *BMC Geriatr.* 23:246. 10.1186/s12877-023-03972-5 37098501 PMC10127029

[B86] Kim H. Noh J. Noh Y. Oh S. S. Koh S. B. Kim C. (2019). Gender difference in the effects of outdoor air pollution on cognitive function among elderly in Korea. *Front. Public Health* 7:375. 10.3389/fpubh.2019.00375 31921740 PMC6915851

[B87] Knopman D. S. Jones D. T. Greicius M. D. (2021). Failure to demonstrate efficacy of aducanumab: an analysis of the EMERGE and ENGAGE trials as reported by Biogen, December 2019. *Alzheimers Dement.* 17 696–701. 10.1002/alz.12213 33135381

[B88] Knouse M. C. McGrath A. G. Deutschmann A. U. Rich M. T. Zallar L. J. Rajadhyaksha A. M. (2022). Sex differences in the medial prefrontal cortical glutamate system. *Biol. Sex Differ.* 13:66. 10.1186/s13293-022-00468-6 36348414 PMC9641904

[B89] Kodama L. Guzman E. Etchegaray J. I. Li Y. Sayed F. A. Zhou L. (2020). Microglial microRNAs mediate sex-specific responses to tau pathology. *Nat. Neurosci.* 23 167–171. 10.1038/s41593-019-0560-7 31873194 PMC7394069

[B90] Kovács Z. Brunner B. D’Agostino D. P. Ari C. (2020). Age- and sex-dependent modulation of exogenous ketone supplement-evoked effects on blood glucose and ketone body levels in Wistar albino Glaxo Rijswijk rats. *Front. Neurosci.* 14:618422. 10.3389/fnins.2020.618422 33505242 PMC7829499

[B91] Krestova M. Ricny J. Bartos A. (2018). Changes in concentrations of tau-reactive antibodies are dependent on sex in Alzheimer’s disease patients. *J. Neuroimmunol.* 322 1–8. 10.1016/j.jneuroim.2018.05.004 29789140

[B92] Kritzer M. F. Creutz L. M. (2008). Region and sex differences in constituent dopamine neurons and immunoreactivity for intracellular estrogen and androgen receptors in mesocortical projections in rats. *J. Neurosci.* 28 9525–9535. 10.1523/JNEUROSCI.2637-08.2008 18799684 PMC2613180

[B93] Kuller L. H. Lopez O. L. (2021). ENGAGE and EMERGE: truth and consequences? *Alzheimers Dement.* 17 692–695. 10.1002/alz.12286 33656288 PMC8248059

[B94] Kurkinen M. (2023). Lecanemab (Leqembi) is not the right drug for patients with Alzheimer’s disease. *Adv. Clin. Exp. Med.* 32 943–947. 10.17219/acem/171379 37676096

[B95] Kwon M. Jung J. Yu H. Lee D. (2017). HIDEEP: a systems approach to predict hormone impacts on drug efficacy based on effect paths. *Sci. Rep.* 7:16600. 10.1038/s41598-017-16855-8 29192270 PMC5709390

[B96] Landucci E. Mazzantini C. Lana D. Calvani M. Magni G. Giovannini M. G. (2022). Cannabidiol inhibits microglia activation and mitigates neuronal damage induced by kainate in an in-vitro seizure model. *Neurobiol. Dis.* 174:105895. 10.1016/j.nbd.2022.105895 36240948

[B97] Lassek W. D. Gaulin S. J. (2011). Sex differences in the relationship of dietary Fatty acids to cognitive measures in American children. *Front. Evol. Neurosci.* 3:5. 10.3389/fnevo.2011.00005 22065957 PMC3206402

[B98] Lee C. H. Lui D. T. W. Cheung C. Y. Y. Woo Y. C. Fong C. H. Y. Yuen M. M. A. (2021). Different glycaemia-related risk factors for incident Alzheimer’s disease in men and women with type 2 diabetes-A sex-specific analysis of the Hong Kong diabetes database. *Diabetes Metab. Res. Rev.* 37:e3401. 10.1002/dmrr.3401 32870568

[B99] Lee J. K. Kim N. J. (2017). Recent advances in the inhibition of p38 MAPK as a potential strategy for the treatment of Alzheimer’s disease. *Molecules* 22:1287. 10.3390/molecules22081287 28767069 PMC6152076

[B100] Letenneur L. Launer L. J. Andersen K. Dewey M. E. Ott A. Copeland J. R. (2000). Education and the risk for Alzheimer’s disease: sex makes a difference. EURODEM pooled analyses. EURODEM incidence research group. *Am. J. Epidemiol.* 151 1064–1071. 10.1093/oxfordjournals.aje.a010149 10873130

[B101] Lewcock J. W. Schlepckow K. Di Paolo G. Tahirovic S. Monroe K. M. Haass C. (2020). Emerging microglia biology defines novel therapeutic approaches for Alzheimer’s disease. *Neuron* 108 801–821. 10.1016/j.neuron.2020.09.029 33096024

[B102] Li G. D. Bi R. Zhang D. F. Xu M. Luo R. Wang D. (2017). Female-specific effect of the BDNF gene on Alzheimer’s disease. *Neurobiol. Aging* 53 192.e11–192.e19. 10.1016/j.neurobiolaging.2016.12.023 28202203

[B103] Li M. Zhao J. Tang Q. Zhang Q. Wang Y. Zhang J. (2021). Lamivudine improves cognitive decline in SAMP8 mice: integrating in vivo pharmacological evaluation and network pharmacology. *J. Cell. Mol. Med.* 25 8490–8503. 10.1111/jcmm.16811 34374199 PMC8419189

[B104] Livingston G. Huntley J. Sommerlad A. Ames D. Ballard C. Banerjee S. (2020). Dementia prevention, intervention, and care: 2020 report of the Lancet Commission. *Lancet* 396 413–446. 10.1016/S0140-6736(20)30367-6 32738937 PMC7392084

[B105] Luchsinger J. A. Palta P. Rippon B. Soto L. Ceballos F. Pardo M. (2020). Sex differences in in vivo Alzheimer’s disease neuropathology in late middle-aged hispanics. *J. Alzheimers Dis.* 74 1243–1252. 10.3233/JAD-191183 32250303 PMC7656318

[B106] Lynch M. A. (2022). Exploring sex-related differences in microglia may be a game-changer in precision medicine. *Front. Aging Neurosci.* 14:868448. 10.3389/fnagi.2022.868448 35431903 PMC9009390

[B107] Ma Y. Ajnakina O. Steptoe A. Cadar D. (2020). Higher risk of dementia in English older individuals who are overweight or obese. *Int. J. Epidemiol.* 49 1353–1365. 10.1093/ije/dyaa099 32575116 PMC7660153

[B108] Maccora J. Peters R. Anstey K. J. (2020). What does (low) education mean in terms of dementia risk? A systematic review and meta-analysis highlighting inconsistency in measuring and operationalising education. *SSM Popul. Health* 12:100654. 10.1016/j.ssmph.2020.100654 33313373 PMC7721642

[B109] MacGowan S. H. Wilcock G. K. Scott M. (1998). Effect of gender and apolipoprotein E genotype on response to anticholinesterase therapy in Alzheimer’s disease. *Int. J. Geriatr. Psychiatry* 13 625–630. 9777427 10.1002/(sici)1099-1166(199809)13:9<625::aid-gps835>3.0.co;2-2

[B110] MacPherson K. P. Sompol P. Kannarkat G. T. Chang J. Sniffen L. Wildner M. E. (2017). Peripheral administration of the soluble TNF inhibitor XPro1595 modifies brain immune cell profiles, decreases beta-amyloid plaque load, and rescues impaired long-term potentiation in 5xFAD mice. *Neurobiol. Dis.* 102 81–95. 10.1016/j.nbd.2017.02.010 28237313 PMC5464789

[B111] Malén T. Karjalainen T. Isojärvi J. Vehtari A. Bürkner P. C. Putkinen V. (2022). Atlas of type 2 dopamine receptors in the human brain: age and sex dependent variability in a large PET cohort. *Neuroimage* 255:119149. 10.1016/j.neuroimage.2022.119149 35367652

[B112] Manji Z. Rojas A. Wang W. Dingledine R. Varvel N. H. Ganesh T. (2019). 5xFAD mice display sex-dependent inflammatory gene induction during the prodromal stage of Alzheimer’s disease. *J. Alzheimers Dis.* 70 1259–1274. 10.3233/JAD-180678 31322556 PMC7379326

[B113] Mao Y. F. Guo Z. Zheng T. Jiang Y. Yan Y. Yin X. (2016). Intranasal insulin alleviates cognitive deficits and amyloid pathology in young adult APPswe/PS1dE9 mice. *Aging Cell* 15 893–902. 10.1111/acel.12498 27457264 PMC5013027

[B114] Martinez-Coria H. Green K. N. Billings L. M. Kitazawa M. Albrecht M. Rammes G. (2010). Memantine improves cognition and reduces Alzheimer’s-like neuropathology in transgenic mice. *Am. J. Pathol.* 176 870–880. 10.2353/ajpath.2010.090452 20042680 PMC2808092

[B115] Martinkova J. Quevenco F. C. Karcher H. Ferrari A. Sandset E. C. Szoeke C. (2021). Proportion of women and reporting of outcomes by sex in clinical trials for Alzheimer disease: a systematic review and meta-analysis. *JAMA Netw. Open* 4:e2124124. 10.1001/jamanetworkopen.2021.24124 34515784 PMC12243614

[B116] Matsushita T. Otani K. Yoshiga M. Hirano M. Noda K. Kurosaka D. (2023). Inhibitory effect of baricitinib on microglia and STAT3 in a region with a weak blood–brain barrier in a mouse model of rheumatoid arthritis. *Rheumatology (Oxford)* 62 2908–2917. 10.1093/rheumatology/kead013 36648313

[B117] Meadowcroft M. D. Connor J. R. Yang Q. X. (2015). Cortical iron regulation and inflammatory response in Alzheimer’s disease and APPSWE/PS1DeltaE9 mice: a histological perspective. *Front. Neurosci.* 9:255. 10.3389/fnins.2015.00255 26257600 PMC4511841

[B118] Mehta N. Rodrigues C. Lamba M. Wu W. Bronskill S. E. Herrmann N. (2017). Systematic review of sex-specific reporting of data: cholinesterase inhibitor example. *J. Am. Geriatr. Soc.* 65 2213–2219. 10.1111/jgs.15020 28832937

[B119] Meilandt W. J. Ngu H. Gogineni A. Lalehzadeh G. Lee S. H. Srinivasan K. (2020). Trem2 deletion reduces late-stage amyloid plaque accumulation, elevates the Abeta42:Abeta40 ratio, and exacerbates axonal dystrophy and dendritic spine loss in the PS2APP Alzheimer’s mouse model. *J. Neurosci.* 40 1956–1974. 10.1523/JNEUROSCI.1871-19.2019 31980586 PMC7046459

[B120] Mela V. Sayd Gaban A. O’Neill E. Bechet S. Walsh A. Lynch M. A. (2022). The modulatory effects of DMF on microglia in aged mice are sex-specific. *Cells* 11:729. 10.3390/cells11040729 35203379 PMC8870377

[B121] Mielke M. M. (2018). Sex and gender differences in Alzheimer’s disease dementia. *Psychiatr. Times* 35 14–17. 30820070 PMC6390276

[B122] Mijailović N. R. Vesic K. Arsenijevic D. Milojević-Rakić M. Borovcanin M. M. (2022). Galectin-3 involvement in cognitive processes for new therapeutic considerations. *Front. Cell. Neurosci.* 16:923811. 10.3389/fncel.2022.923811 35875353 PMC9296991

[B123] Mintun M. A. Lo A. C. Duggan Evans C. Wessels A. M. Ardayfio P. A. Andersen S. W. (2021). Donanemab in early Alzheimer’s disease. *N. Engl. J. Med.* 384 1691–1704. 10.1056/NEJMoa2100708 33720637

[B124] Mishra A. Brinton R. D. (2018). Inflammation: bridging age, menopause and APOEepsilon4 genotype to Alzheimer’s disease. *Front. Aging Neurosci.* 10:312. 10.3389/fnagi.2018.00312 30356809 PMC6189518

[B125] Mishra A. Wang Y. Yin F. Vitali F. Rodgers K. E. Soto M. (2022). A tale of two systems: lessons learned from female mid-life aging with implications for Alzheimer’s prevention and treatment. *Ageing Res. Rev.* 74:101542. 10.1016/j.arr.2021.101542 34929348 PMC8884386

[B126] Misrani A. Tabassum S. Yang L. (2021). Mitochondrial dysfunction and oxidative stress in Alzheimer’s disease. *Front. Aging Neurosci.* 13:617588. 10.3389/fnagi.2021.617588 33679375 PMC7930231

[B127] Moen J. K. Lee A. M. (2021). Sex differences in the nicotinic acetylcholine receptor system of rodents: impacts on nicotine and alcohol reward behaviors. *Front. Neurosci.* 15:745783. 10.3389/fnins.2021.745783 34621155 PMC8490611

[B128] Monteiro K. L. C. Dos Santos Alcântara M. G. Freire N. M. L. Brandão E. M. do Nascimento V. L. Dos Santos Viana L. M. (2023). BACE-1 inhibitors targeting Alzheimer’s disease. *Curr. Alzheimer Res.* 20 131–148. 10.2174/1567205020666230612155953 37309767

[B129] Morera C. Sabates S. Jaen A. (2015). Sex differences in N-palmitoylethanolamide effectiveness in neuropathic pain associated with lumbosciatalgia. *Pain Manag.* 5 81–87. 10.2217/pmt.15.5 25806902

[B130] Mosconi L. Brinton R. D. (2018). How would we combat menopause as an Alzheimer’s risk factor? *Expert Rev. Neurother.* 18 689–691. 10.1080/14737175.2018.1510320 30091648 PMC6296216

[B131] Mosconi L. Rahman A. Diaz I. Wu X. Scheyer O. Hristov H. W. (2018). Increased Alzheimer’s risk during the menopause transition: a 3-year longitudinal brain imaging study. *PLoS One* 13:e0207885. 10.1371/journal.pone.0207885 30540774 PMC6291073

[B132] Mulvey B. Bhatti D. L. Gyawali S. Lake A. M. Kriaucionis S. Ford C. P. (2018). Molecular and functional sex differences of noradrenergic neurons in the mouse locus coeruleus. *Cell Rep.* 23 2225–2235. 10.1016/j.celrep.2018.04.054 29791834 PMC6070358

[B133] Nebel R. A. Aggarwal N. T. Barnes L. L. Gallagher A. Goldstein J. M. Kantarci K. (2018). Understanding the impact of sex and gender in Alzheimer’s disease: a call to action. *Alzheimers Dement.* 14 1171–1183. 10.1016/j.jalz.2018.04.008 29907423 PMC6400070

[B134] Neu S. C. Pa J. Kukull W. Beekly D. Kuzma A. Gangadharan P. (2017). Apolipoprotein E genotype and sex risk factors for Alzheimer disease: a meta-analysis. *JAMA Neurol.* 74 1178–1189. 10.1001/jamaneurol.2017.2188 28846757 PMC5759346

[B135] Nishizawa S. Benkelfat C. Young S. N. Leyton M. Mzengeza S. de Montigny C. (1997). Differences between males and females in rates of serotonin synthesis in human brain. *Proc. Natl. Acad. Sci. U.S.A.* 94 5308–5313. 10.1073/pnas.94.10.5308 9144233 PMC24674

[B136] Nørgaard C. H. Friedrich S. Hansen C. T. Gerds T. Ballard C. Møller D. V. (2022). Treatment with glucagon-like peptide-1 receptor agonists and incidence of dementia: data from pooled double-blind randomized controlled trials and nationwide disease and prescription registers. *Alzheimers Dement.* 8:e12268. 10.1002/trc2.12268 35229024 PMC8864443

[B137] Normandin M. D. Zheng M. Q. Lin K. S. Mason N. S. Lin S. F. Ropchan J. (2015). Imaging the cannabinoid CB1 receptor in humans with [11C]OMAR: assessment of kinetic analysis methods, test–retest reproducibility, and gender differences. *J. Cereb. Blood Flow Metab.* 35 1313–1322. 10.1038/jcbfm.2015.46 25833345 PMC4528005

[B138] Norton J. Carrière I. Pérès K. Gabelle A. Berr C. Ritchie K. (2019). Sex-specific depressive symptoms as markers of pre-Alzheimer dementia: findings from the Three-City cohort study. *Transl. Psychiatry* 9:291. 10.1038/s41398-019-0620-5 31712553 PMC6848073

[B139] O’Neill E. Mela V. Gaban A. S. Bechet S. McGrath A. Walsh A. (2022). Sex-related microglial perturbation is related to mitochondrial changes in a model of Alzheimer’s disease. *Front. Cell. Neurosci.* 16:939830. 10.3389/fncel.2022.939830 35875349 PMC9297004

[B140] Ohyagi Y. Miyoshi K. Nakamura N. (2019). Therapeutic strategies for Alzheimer’s disease in the view of diabetes mellitus. *Adv. Exp. Med. Biol.* 1128 227–248. 10.1007/978-981-13-3540-2_11 31062332

[B141] Ostrowitzki S. Bittner T. Sink K. M. Mackey H. Rabe C. Honig L. S. (2022). Evaluating the safety and efficacy of crenezumab vs placebo in adults with early Alzheimer disease: two Phase 3 randomized placebo-controlled trials. *JAMA Neurol.* 79 1113–1121. 10.1001/jamaneurol.2022.2909 36121669 PMC9486635

[B142] Oveisgharan S. Arvanitakis Z. Yu L. Farfel J. Schneider J. A. Bennett D. A. (2018). Sex differences in Alzheimer’s disease and common neuropathologies of aging. *Acta Neuropathol.* 136 887–900. 10.1007/s00401-018-1920-1 30334074 PMC6279593

[B143] Özdemir B. C. Gerard C. L. Espinosa da Silva C. (2022). Sex and gender differences in anticancer treatment toxicity: a call for revisiting drug dosing in oncology. *Endocrinology* 163:bqac058. 10.1210/endocr/bqac058 35560216 PMC9113364

[B144] Pa J. Aslanyan V. Casaletto K. B. Rentería M. A. Harrati A. Tom S. E. (2022). Effects of sex, APOE4, and lifestyle activities on cognitive reserve in older adults. *Neurology* 99 e789–e798. 10.1212/WNL.0000000000200675 35858818 PMC9484731

[B145] Paranjpe M. D. Belonwu S. Wang J. K. Oskotsky T. Gupta A. Taubes A. (2021). Sex-specific cross tissue meta-analysis identifies immune dysregulation in women with Alzheimer’s disease. *Front. Aging Neurosci.* 13:735611. 10.3389/fnagi.2021.735611 34658838 PMC8515049

[B146] Park J. C. Lim H. Byun M. S. Yi D. Byeon G. Jung G. (2023). Sex differences in the progression of glucose metabolism dysfunction in Alzheimer’s disease. *Exp. Mol. Med.* 55 1023–1032. 10.1038/s12276-023-00993-3 37121979 PMC10238450

[B147] Patten K. T. Valenzuela A. E. Wallis C. Berg E. L. Silverman J. L. Bein K. J. (2021). The effects of chronic exposure to ambient traffic-related air pollution on Alzheimer’s disease phenotypes in wildtype and genetically predisposed male and female rats. *Environ. Health Perspect.* 129:57005. 10.1289/EHP8905 33971107 PMC8110309

[B148] Pavlidi P. Kokras N. Dalla C. (2021). Antidepressants’ effects on testosterone and estrogens: what do we know? *Eur. J. Pharmacol.* 899:173998. 10.1016/j.ejphar.2021.173998 33676942

[B149] Placanica L. Zhu L. Li Y. M. (2009). Gender- and age-dependent gamma-secretase activity in mouse brain and its implication in sporadic Alzheimer disease. *PLoS One* 4:e5088. 10.1371/journal.pone.0005088 19352431 PMC2661375

[B150] Podcasy J. L. Epperson C. N. (2016). Considering sex and gender in Alzheimer disease and other dementias. *Dialogues Clin. Neurosci.* 18 437–446. 10.31887/DCNS.2016.18.4/cepperson 28179815 PMC5286729

[B151] Prokopenko D. Hecker J. Kirchner R. Chapman B. A. Hoffman O. Mullin K. (2020). Identification of novel Alzheimer’s disease loci using sex-specific family-based association analysis of whole-genome sequence data. *Sci. Rep.* 10:5029. 10.1038/s41598-020-61883-6 PMC7081222 32193444

[B152] Rahman A. Hossen M. A. Chowdhury M. F. I. Bari S. Tamanna N. Sultana S. S. (2023). Aducanumab for the treatment of Alzheimer’s disease: a systematic review. *Psychogeriatrics* 23 512–522. 10.1111/psyg.12944 36775284 PMC11578022

[B153] Rahman A. Jackson H. Hristov H. Isaacson R. S. Saif N. Shetty T. (2019). Sex and gender driven modifiers of Alzheimer’s: the role for estrogenic control across age, race, medical, and lifestyle risks. *Front. Aging Neurosci.* 11:315. 10.3389/fnagi.2019.00315 31803046 PMC6872493

[B154] Raparelli V. Elharram M. Moura C. S. Abrahamowicz M. Bernatsky S. Behlouli H. (2020). Sex differences in cardiovascular effectiveness of newer glucose-lowering drugs added to metformin in type 2 diabetes mellitus. *J. Am. Heart Assoc.* 9:e012940. 10.1161/JAHA.119.012940 31902326 PMC6988160

[B155] Reardon S. (2023b). Alzheimer’s drug trials plagued by lack of racial diversity. *Nature* 620 256–257. 10.1038/d41586-023-02464-1 37532857

[B156] Reardon S. (2023a). Alzheimer’s drug donanemab: what promising trial means for treatments. *Nature* 617 232–233. 10.1038/d41586-023-01537-5 37142723

[B157] Ren Y. Savadlou A. Park S. Siska P. Epp J. R. Sargin D. (2023). The impact of loneliness and social isolation on the development of cognitive decline and Alzheimer’s disease. *Front. Neuroendocrinol.* 69:101061. 10.1016/j.yfrne.2023.101061 36758770

[B158] Rentzeperi E. Pegiou S. Koufakis T. Grammatiki M. Kotsa K. (2022). Sex differences in response to treatment with glucagon-like peptide 1 receptor agonists: opportunities for a tailored approach to diabetes and obesity care. *J. Pers. Med.* 12:454. 10.3390/jpm12030454 35330453 PMC8950819

[B159] Riedel B. C. Thompson P. M. Brinton R. D. (2016). Age, APOE and sex: triad of risk of Alzheimer’s disease. *J. Steroid Biochem. Mol. Biol.* 160 134–147. 10.1016/j.jsbmb.2016.03.012 26969397 PMC4905558

[B160] Rigaud A. S. Traykov L. Caputo L. Guelfi M. C. Latour F. Couderc R. (2000). The apolipoprotein E epsilon4 allele and the response to tacrine therapy in Alzheimer’s disease. *Eur. J. Neurol.* 7 255–258. 10.1046/j.1468-1331.2000.00073.x 10886308

[B161] Rivera F. B. Tang V. A. S. De Luna D. V. Lerma E. V. Vijayaraghavan K. Kazory A. (2023). Sex differences in cardiovascular outcomes of SGLT-2 inhibitors in heart failure randomized controlled trials: a systematic review and meta-analysis. *Am. Heart J. Plus Cardiol. Res. Pract.* 26:100261. 10.1016/j.ahjo.2023.100261 37305172 PMC10256233

[B162] Roberts M. Sevastou I. Imaizumi Y. Mistry K. Talma S. Dey M. (2020). Pre-clinical characterisation of E2814, a high-affinity antibody targeting the microtubule-binding repeat domain of tau for passive immunotherapy in Alzheimer’s disease. *Acta Neuropathol. Commun.* 8:13. 10.1186/s40478-020-0884-2 32019610 PMC7001291

[B163] Rubino T. Parolaro D. (2011). Sexually dimorphic effects of cannabinoid compounds on emotion and cognition. *Front. Behav. Neurosci.* 5:64. 10.3389/fnbeh.2011.00064 21991251 PMC3181427

[B164] Ruiz F. Keeley A. Leglise P. Tuleu C. Lachuer C. Rwabihama J. P. (2019). Sex differences in medicine acceptability: a new factor to be considered in medicine formulation. *Pharmaceutics* 11:368. 10.3390/pharmaceutics11080368 PMC6723034 31374869

[B165] Rynearson K. D. Ponnusamy M. Prikhodko O. Xie Y. Zhang C. Nguyen P. (2021). Preclinical validation of a potent gamma-secretase modulator for Alzheimer’s disease prevention. *J. Exp. Med.* 218:e20202560. 10.1084/jem.20202560 33651103 PMC7931646

[B166] Sabbagh M. N. Cummings J. (2021). Open Peer commentary to “Failure to demonstrate efficacy of aducanumab: an analysis of the EMERGE and ENGAGE Trials as reported by Biogen December 2019”. *Alzheimers Dement.* 17 702–703. 10.1002/alz.12235 33135288 PMC8043968

[B167] Sahlholm K. Liao F. Holtzman D. M. Xu J. Mach R. H. (2015). Sigma-2 receptor binding is decreased in female, but not male, APP/PS1 mice. *Biochem. Biophys. Res. Commun.* 460 439–445. 10.1016/j.bbrc.2015.03.052 25796326 PMC6818091

[B168] Sanfilippo C. Castrogiovanni P. Imbesi R. Kazakowa M. Musumeci G. Blennow K. (2019). Sex difference in CHI3L1 expression levels in human brain aging and in Alzheimer’s disease. *Brain Res.* 1720:146305. 10.1016/j.brainres.2019.146305 31247206

[B169] Sárvári M. Hrabovszky E. Kalló I. Solymosi N. Likó I. Berchtold N. (2012). Menopause leads to elevated expression of macrophage-associated genes in the aging frontal cortex: rat and human studies identify strikingly similar changes. *J. Neuroinflammation* 9:264. 10.1186/1742-2094-9-264 23206327 PMC3558453

[B170] Scacchi R. Gambina G. Broggio E. Corbo R. M. (2014). Sex and ESR1 genotype may influence the response to treatment with donepezil and rivastigmine in patients with Alzheimer’s disease. *Int. J. Geriatr. Psychiatry* 29 610–615. 10.1002/gps.4043 24150894

[B171] Schneider A. L. C. Selvin E. Latour L. Turtzo L. C. Coresh J. Mosley T. (2021). Head injury and 25-year risk of dementia. *Alzheimers Dement.* 17 1432–1441. 10.1002/alz.12315 33687142 PMC9422954

[B172] Schneider L. S. (2022). Editorial: aducanumab trials EMERGE but don’t ENGAGE. *J. Prev. Alzheimers Dis.* 9 193–196. 10.14283/jpad.2022.37 35542990

[B173] Schwartz J. B. Weintraub S. (2021). Treatment for Alzheimer disease-sex and gender effects need to be explicitly analyzed and reported in clinical trials. *JAMA Netw. Open* 4:e2124386. 10.1001/jamanetworkopen.2021.24386 34515788

[B174] Sharon A. Erez H. Spira M. E. (2022). Significant sex differences in the efficacy of the CSF1R inhibitor-PLX5622 on rat brain microglia elimination. *Pharmaceuticals* 15:569. 10.3390/ph15050569 35631395 PMC9145577

[B175] Sheng X. Yao Y. Huang R. Xu Y. Zhu Y. Chen L. (2021). Identification of the minimal active soluble TREM2 sequence for modulating microglial phenotypes and amyloid pathology. *J. Neuroinflammation* 18:286. 10.1186/s12974-021-02340-7 34893068 PMC8665564

[B176] Shi L. Steenland K. Li H. Liu P. Zhang Y. Lyles R. H. (2021). A national cohort study (2000–2018) of long-term air pollution exposure and incident dementia in older adults in the United States. *Nat. Commun.* 12:6754. 10.1038/s41467-021-27049-2 34799599 PMC8604909

[B177] Shokouhi S. Taylor W. D. Albert K. Kang H. Newhouse P. A. Alzheimer’s Disease (2020). In vivo network models identify sex differences in the spread of tau pathology across the brain. *Alzheimers Dement.* 12:e12016. 10.1002/dad2.12016 32280740 PMC7144772

[B178] Sims J. R. Zimmer J. A. Evans C. D. Lu M. Ardayfio P. Sparks J. (2023). Donanemab in early symptomatic Alzheimer disease: the TRAILBLAZER-ALZ 2 randomized clinical trial. *JAMA* 330 512–527. 10.1001/jama.2023.13239 37459141 PMC10352931

[B179] Sindi S. Kåreholt I. Ngandu T. Rosenberg A. Kulmala J. Johansson L. (2021). Sex differences in dementia and response to a lifestyle intervention: evidence from Nordic population-based studies and a prevention trial. *Alzheimers Dement.* 17 1166–1178. 10.1002/alz.12279 34255432 PMC8361986

[B180] Smith C. J. W. Ratnaseelan A. M. Veenema A. H. (2018). Robust age, but limited sex, differences in mu-opioid receptors in the rat brain: relevance for reward and drug-seeking behaviors in juveniles. *Brain Struct. Funct.* 223 475–488. 10.1007/s00429-017-1498-8 28871491 PMC5772146

[B181] Smith E. S. Jonason A. Reilly C. Veeraraghavan J. Fisher T. Doherty M. (2015). SEMA4D compromises blood–brain barrier, activates microglia, and inhibits remyelination in neurodegenerative disease. *Neurobiol. Dis.* 73 254–268. 10.1016/j.nbd.2014.10.008 25461192

[B182] Song C. Shi J. Zhang P. Zhang Y. Xu J. Zhao L. (2022). Immunotherapy for Alzheimer’s disease: targeting beta-amyloid and beyond. *Transl. Neurodegener.* 11:18. 10.1186/s40035-022-00292-3 35300725 PMC8932191

[B183] Song Q. H. Zhao K. X. Huang S. Chen T. He L. (2024). Escape from X-chromosome inactivation and sex differences in Alzheimer’s disease. *Rev. Neurosci.* 10.1515/revneuro-2023-0108 [Epub ahead of print]. 38157427

[B184] Songtachalert T. Roomruangwong C. Carvalho A. F. Bourin M. Maes M. (2018). Anxiety disorders: sex differences in serotonin and tryptophan metabolism. *Curr. Top. Med. Chem.* 18 1704–1715. 10.2174/1568026618666181115093136 30430940

[B185] Sramek J. J. Murphy M. F. Cutler N. R. (2016). Sex differences in the psychopharmacological treatment of depression. *Dialogues Clin. Neurosci.* 18 447–457. 10.31887/DCNS.2016.18.4/ncutler 28179816 PMC5286730

[B186] Stephen T. L. Cacciottolo M. Balu D. Morgan T. E. LaDu M. J. Finch C. E. (2019). APOE genotype and sex affect microglial interactions with plaques in Alzheimer’s disease mice. *Acta Neuropathol. Commun.* 7:82. 10.1186/s40478-019-0729-z 31113487 PMC6528326

[B187] Stonehouse W. Conlon C. A. Podd J. Hill S. R. Minihane A. M. Haskell C. (2013). DHA supplementation improved both memory and reaction time in healthy young adults: a randomized controlled trial. *Am. J. Clin. Nutr.* 97 1134–1143. 10.3945/ajcn.112.053371 23515006

[B188] Strefeler A. Jan M. Quadroni M. Teav T. Rosenberg N. Chatton J. Y. (2023). Molecular insights into sex-specific metabolic alterations in Alzheimer’s mouse brain using multi-omics approach. *Alzheimers Res. Ther.* 15:8. 10.1186/s13195-023-01162-4 36624525 PMC9827669

[B189] Strittmatter W. J. Saunders A. M. Schmechel D. Pericak-Vance M. Enghild J. Salvesen G. S. (1993). Apolipoprotein E: high-avidity binding to beta-amyloid and increased frequency of type 4 allele in late-onset familial Alzheimer disease. *Proc. Natl. Acad. Sci. U.S.A.* 90 1977–1981. 10.1073/pnas.90.5.1977 8446617 PMC46003

[B190] Sun P. Wang J. Zhang M. Duan X. Wei Y. Xu F. (2020). Sex-related differential whole-brain input atlas of locus coeruleus noradrenaline neurons. *Front. Neural Circuits* 14:53. 10.3389/fncir.2020.00053 33071759 PMC7541090

[B191] Swanson C. J. Zhang Y. Dhadda S. Wang J. Kaplow J. Lai R. Y. K. (2021). A randomized, double-blind, phase 2b proof-of-concept clinical trial in early Alzheimer’s disease with lecanemab, an anti-Abeta protofibril antibody. *Alzheimers Res. Ther.* 13:80. 10.1186/s13195-021-00813-8 33865446 PMC8053280

[B192] Swerdlow R. H. (2018). Mitochondria and mitochondrial cascades in Alzheimer’s disease. *J. Alzheimers Dis.* 62 1403–1416. 10.3233/JAD-170585 29036828 PMC5869994

[B193] Tai S. Y. Shen C. T. Wang L. F. Chien C. Y. (2021). Association of sudden sensorineural hearing loss with dementia: a nationwide cohort study. *BMC Neurol.* 21:88. 10.1186/s12883-021-02106-x 33627087 PMC7904508

[B194] Taki Y. Kinomura S. Sato K. Inoue K. Goto R. Okada K. (2008). Relationship between body mass index and gray matter volume in 1,428 healthy individuals. *Obesity* 16 119–124. 10.1038/oby.2007.4 18223623

[B195] Tan Y. Zheng Y. Xu D. Sun Z. Yang H. Yin Q. (2021). Galectin-3: a key player in microglia-mediated neuroinflammation and Alzheimer’s disease. *Cell Biosci.* 11:78. 10.1186/s13578-021-00592-7 33906678 PMC8077955

[B196] Taubøll E. Isojärvi J. I. T. Herzog A. G. (2021). The interactions between reproductive hormones and epilepsy. *Handb. Clin. Neurol.* 182 155–174. 10.1016/B978-0-12-819973-2.00011-3 34266590

[B197] Teich A. F. Sharma E. Barnwell E. Zhang H. Staniszewski A. Utsuki T. (2018). Translational inhibition of APP by Posiphen: efficacy, pharmacodynamics, and pharmacokinetics in the APP/PS1 mouse. *Alzheimers Dement.* 4 37–45. 10.1016/j.trci.2017.12.001 29955650 PMC6021259

[B198] Tenkorang M. A. Snyder B. Cunningham R. L. (2018). Sex-related differences in oxidative stress and neurodegeneration. *Steroids* 133 21–27. 10.1016/j.steroids.2017.12.010 29274405 PMC5864532

[B199] Ungar L. Altmann A. Greicius M. D. (2014). Apolipoprotein E, gender, and Alzheimer’s disease: an overlooked, but potent and promising interaction. *Brain Imaging Behav.* 8 262–273. 10.1007/s11682-013-9272-x 24293121 PMC4282773

[B200] Valera E. Mante M. Anderson S. Rockenstein E. Masliah E. (2015). Lenalidomide reduces microglial activation and behavioral deficits in a transgenic model of Parkinson’s disease. *J. Neuroinflammation* 12:93. 10.1186/s12974-015-0320-x 25966683 PMC4432827

[B201] van Dyck C. H. Swanson C. J. Aisen P. Bateman R. J. Chen C. Gee M. (2023). Lecanemab in early Alzheimer’s disease. *N. Engl. J. Med.* 388 9–21. 10.1056/NEJMoa2212948 36449413

[B202] Vergallo A. Houot M. Cavedo E. Lemercier P. Vanmechelen E. De Vos A. (2019). Brain Abeta load association and sexual dimorphism of plasma BACE1 concentrations in cognitively normal individuals at risk for AD. *Alzheimers Dement.* 15 1274–1285. 10.1016/j.jalz.2019.07.001 31627825

[B203] Verhagen C. Janssen J. Biessels G. J. Johansen O. E. Exalto L. G. (2022). Females with type 2 diabetes are at higher risk for accelerated cognitive decline than males: CAROLINA-COGNITION study. *Nutr. Metab. Cardiovasc. Dis.* 32 355–364. 10.1016/j.numecd.2021.10.013 34895804

[B204] Viña J. Lloret A. (2010). Why women have more Alzheimer’s disease than men: gender and mitochondrial toxicity of amyloid-beta peptide. *J. Alzheimers Dis.* 20 (Suppl. 2) S527–S533. 10.3233/JAD-2010-100501 20442496

[B205] Viswanathan J. Mäkinen P. Helisalmi S. Haapasalo A. Soininen H. Hiltunen M. (2009). An association study between granulin gene polymorphisms and Alzheimer’s disease in Finnish population. *Am. J. Med. Genet. Part B Neuropsychiatr. Genet.* 150B 747–750. 10.1002/ajmg.b.30889 19016491

[B206] Wang R. H. Bejar C. Weinstock M. (2000). Gender differences in the effect of rivastigmine on brain cholinesterase activity and cognitive function in rats. *Neuropharmacology* 39 497–506. 10.1016/s0028-3908(99)00157-4 10698015

[B207] Wang S. Sudan R. Peng V. Zhou Y. Du S. Yuede C. M. (2022). TREM2 drives microglia response to amyloid-beta via SYK-dependent and -independent pathways. *Cell* 185 4153–4169.e19. 10.1016/j.cell.2022.09.033 36306735 PMC9625082

[B208] Wang W. Zhao F. Ma X. Perry G. Zhu X. (2020). Mitochondria dysfunction in the pathogenesis of Alzheimer’s disease: recent advances. *Mol. Neurodegener.* 15:30. 10.1186/s13024-020-00376-6 32471464 PMC7257174

[B209] Wang X. Broce I. Deters K. D. Fan C. C. Banks S. J. (2022). Identification of sex-specific genetic variants associated with tau PET. *Neurol. Genet.* 8:e200043. 10.1212/NXG.0000000000200043 36530928 PMC9756308

[B210] Wattmo C. Wallin A. K. Londos E. Minthon L. (2011). Predictors of long-term cognitive outcome in Alzheimer’s disease. *Alzheimers Res. Ther.* 3:23. 10.1186/alzrt85 21774798 PMC3226278

[B211] Wickens M. M. Bangasser D. A. Briand L. A. (2018). Sex differences in psychiatric disease: a focus on the glutamate system. *Front. Mol. Neurosci.* 11:197. 10.3389/fnmol.2018.00197 29922129 PMC5996114

[B212] Wilbert-Lampen U. Seliger C. Trapp A. Straube F. Plasse A. (2005). Female sex hormones decrease constitutive endothelin-1 release via endothelial sigma-1/cocaine receptors: an action independent of the steroid hormone receptors. *Endothelium* 12 185–191. 10.1080/10623320500227275 16162441

[B213] Williams O. O. F. Coppolino M. George S. R. Perreault M. L. (2021). Sex differences in dopamine receptors and relevance to neuropsychiatric disorders. *Brain Sci.* 11:1199. 10.3390/brainsci11091199 34573220 PMC8469878

[B214] Xie J. Van Hoecke L. Vandenbroucke R. E. (2021). The impact of systemic inflammation on Alzheimer’s disease pathology. *Front. Immunol.* 12:796867. 10.3389/fimmu.2021.796867 35069578 PMC8770958

[B215] Yaffe K. Haan M. Byers A. Tangen C. Kuller L. (2000). Estrogen use, APOE, and cognitive decline: evidence of gene-environment interaction. *Neurology* 54 1949–1954. 10.1212/wnl.54.10.1949 10822435

[B216] Yan Y. Dominguez S. Fisher D. W. Dong H. (2018). Sex differences in chronic stress responses and Alzheimer’s disease. *Neurobiol. Stress* 8 120–126. 10.1016/j.ynstr.2018.03.002 29888307 PMC5991323

[B217] Ye L. Sun Y. Jiang Z. Wang G. (2021). L-serine, an endogenous amino acid, is a potential neuroprotective agent for neurological disease and injury. *Front. Mol. Neurosci.* 14:726665. 10.3389/fnmol.2021.726665 34552468 PMC8450333

[B218] Yiannopoulou K. G. Anastasiou A. I. Zachariou V. Pelidou S. (2019). Reasons for failed trials of disease-modifying treatments for Alzheimer disease and their contribution in recent research. *Biomedicines* 7:97. 10.3390/biomedicines7040097 31835422 PMC6966425

[B219] Yoshida T. Kuwabara Y. Sasaki M. Fukumura T. Ichimiya A. Takita M. (2000). Sex-related differences in the muscarinic acetylcholinergic receptor in the healthy human brain—A positron emission tomography study. *Ann. Nucl. Med.* 14 97–101. 10.1007/BF02988587 10830526

[B220] Youness A. Miquel C. H. Guéry J. C. (2021). Escape from X chromosome inactivation and the female predominance in autoimmune diseases. *Int. J. Mol. Sci.* 22:1114. 10.3390/ijms22031114 33498655 PMC7865432

[B221] Zagorski K. King O. Hovakimyan A. Petrushina I. Antonyan T. Chailyan G. (2023). Novel vaccine against pathological pyroglutamate-modified amyloid beta for prevention of Alzheimer’s disease. *Int. J. Mol. Sci.* 24:9797. 10.3390/ijms24129797 37372944 PMC10298272

[B222] Zhang N. Ranson J. M. Zheng Z. J. Hannon E. Zhou Z. Kong X. (2021). Interaction between genetic predisposition, smoking, and dementia risk: a population-based cohort study. *Sci. Rep.* 11:12953. 10.1038/s41598-021-92304-x 34155245 PMC8217565

[B223] Zhao R. Hu W. Tsai J. Li W. Gan W. B. (2017). Microglia limit the expansion of beta-amyloid plaques in a mouse model of Alzheimer’s disease. *Mol. Neurodegener.* 12:47. 10.1186/s13024-017-0188-6 28606182 PMC5468952

[B224] Zhong L. Xu Y. Zhuo R. Wang T. Wang K. Huang R. (2019). Soluble TREM2 ameliorates pathological phenotypes by modulating microglial functions in an Alzheimer’s disease model. *Nat. Commun.* 10:1365. 10.1038/s41467-019-09118-9 30911003 PMC6433910

[B225] Zhou C. Dong C. Xie Z. Hao W. Fu C. Sun H. (2023). Sex-specific associations between diabetes and dementia: the role of age at onset of disease, insulin use and complications. *Biol. Sex Differ.* 14:9. 10.1186/s13293-023-00491-1 36804018 PMC9940390

